# Treatment of Parkinson’s disease with biologics that penetrate the blood–brain barrier via receptor-mediated transport

**DOI:** 10.3389/fnagi.2023.1276376

**Published:** 2023-11-13

**Authors:** William M. Pardridge

**Affiliations:** University of California, Los Angeles, Los Angeles, CA, United States

**Keywords:** blood–brain barrier, Parkinson’s disease, drug delivery, neurotrophins, decoy receptor

## Abstract

Parkinson’s disease (PD) is characterized by neurodegeneration of nigral-striatal neurons in parallel with the formation of intra-neuronal α-synuclein aggregates, and these processes are exacerbated by neuro-inflammation. All 3 components of PD pathology are potentially treatable with biologics. Neurotrophins, such as glial derived neurotrophic factor or erythropoietin, can promote neural repair. Therapeutic antibodies can lead to disaggregation of α-synuclein neuronal inclusions. Decoy receptors can block the activity of pro-inflammatory cytokines in brain. However, these biologic drugs do not cross the blood–brain barrier (BBB). Biologics can be made transportable through the BBB following the re-engineering of the biologic as an IgG fusion protein, where the IgG domain targets an endogenous receptor-mediated transcytosis (RMT) system within the BBB, such as the insulin receptor or transferrin receptor. The receptor-specific antibody domain of the fusion protein acts as a molecular Trojan horse to ferry the biologic into brain via the BBB RMT pathway. This review describes the re-engineering of all 3 classes of biologics (neurotrophins, decoy receptor, therapeutic antibodies) for BBB delivery and treatment of PD. Targeting the RMT pathway at the BBB also enables non-viral gene therapy of PD using lipid nanoparticles (LNP) encapsulated with plasmid DNA encoding therapeutic genes. The surface of the lipid nanoparticle is conjugated with a receptor-specific IgG that triggers RMT of the LNP across the BBB *in vivo*.

## 1. Introduction

Parkinson’s disease (PD) is a severe neurodegenerative condition causing motor and cognitive impairment. The World Health Organization finds the prevalence of PD world-wide has doubled in the last 25 years with 8.5 million cases in 2019, and that the disability-adjusted life years has increased 81% since 2000 (McFarthing et al., 2023). The primary treatment of PD is L-dihydroxyphenylalanine, or L-DOPA, which was approved by the FDA for PD over 50 years ago (Hornykiewicz, 1966). L-DOPA treatment has side effects and nearly half of PD patients develop dyskinesia within 5 years of therapy (Turcano et al., 2018). Given the limitations of L-DOPA therapy in PD, one could ask why this drug has not been supplanted, over the course of the last 50 years, by improved treatments of PD. The answer is the blood–brain barrier (BBB). The viewpoint that the BBB is the limiting factor in PD drug development is derived from 2 considerations. First, approximately 98% of small molecule drugs, and ~ 100% of biologics, do not cross the BBB (Pardridge, 2022a,b). Second, BBB drug delivery is not part of the landscape covering PD drug development as exemplified by the following:

- A PubMed analysis performed July, 2023, using the search term, “Parkinson’s disease treatment,” yields 73,898 citations. However, use of the search term, “Parkinson’s disease treatment and blood-brain barrier drug delivery,” lists 401 citations, or 0.5% of the total.
- A recent review discusses novel therapeutic targets in PD, but makes no mention of BBB drug delivery of new agents developed for PD (Soni et al., 2023). A recent review of 139 clinical trials for PD listed at ClinicalTrials.gov makes no reference to the BBB (McFarthing et al., 2023).
- Therapeutic antibodies do not cross the BBB (Pardridge, 2023a). Yet, several therapeutic antibodies directed against α-synuclein (SYN) entered clinical trials for PD. Last year, 2 such anti-SYN antibodies, prasineuzumab (Pagano et al., 2022) and cinpanemab (Lang et al., 2022) failed to show benefit in PD, but the lack of therapeutic antibody transport across the BBB was not considered in an analysis of the trial failures. An Editorial summarizing these failed clinical trials quoted Churchill, “Success consists of going from failure to failure without loss of enthusiasm” (Whone, 2022).

Rather embracing repetitive failure, the view expressed here is that all future PD drug development needs to explicitly incorporate BBB drug delivery in the overall PD therapeutics program. The irony of this statement is that the efficacy of L-DOPA is, in fact, derived from a BBB drug delivery strategy. L-DOPA is a polar small molecule that should not cross the BBB. However, L-DOPA is a large neutral amino acid (LNAA), and L-DOPA crosses the BBB via a LNAA carrier-mediated transport system (Oldendorf, 1971). The primary LNAA transporter, LAT1 (SLC7A5), was cloned from a rat glioma library (Kanai et al., 1998), and LAT1 is selectively expressed at the BBB (Boado et al., 1999). Cloned LAT1 transports L-DOPA (Kageyama et al., 2000), but does not transport carbidopa (Uchino et al., 2002). Carbidopa, which does not cross the BBB, is formulated as a co-drug with L-DOPA, so as to inhibit peripheral conversion of L-DOPA to dopamine, which is catalyzed by aromatic amino acid decarboxylase (AAAD; Zhu et al., 2017). Subsequent to L-DOPA entry into the brain via BBB transport, the drug is converted to dopamine by cerebral AAAD.

Patients with PD do not present with motor abnormalities prompting L-DOPA therapy until there is greater than 60–80% loss of dopaminergic neurons in the substantia nigra (Masato et al., 2019; Hustad and Aasly, 2020). Therefore, a central goal in PD drug development is pharmacologic intervention that halts or slows the nigral degeneration of dopaminergic neurons. Biologic drugs, such as neurotrophins, decoy receptors, or therapeutic antibodies, are candidates for treatment of the neurodegeneration of PD. However, biologics are large molecule drugs that do not cross the BBB. Biologic drug development for the brain requires the use of a brain drug delivery technology. Brain drug delivery strategies include drug injection into the cerebrospinal fluid (CSF), cerebral implants or convection-enhanced diffusion (CED), trans-nasal drug delivery, nanoparticles, exosomes, and receptor-mediated transcytosis (RMT). These brain drug delivery technologies have been recently reviewed (Pardridge, 2022b).

This review focuses on the re-engineering of biologic agents for PD as pharmaceuticals that penetrate the brain via RMT across the BBB. The biologic agents include neurotrophic factors, such as glial cell line-derived neurotrophic factor (GDNF) or erythropoietin (EPO), decoy receptors that suppress pro-inflammatory cytokines, such as tumor necrosis factor (TNF)-α, and therapeutic monoclonal antibodies (MAb) that either block the formation of α-synuclein aggregates or are neurotrophin receptor agonist antibodies. In each case, the biologic is re-engineered as an IgG fusion protein, where the IgG domain targets an endogenous RMT system at the BBB. These endogenous BBB receptors normally function to enable the RMT of endogenous peptides across the BBB, such as insulin (IR), transferrin (Tf), insulin-like growth factors (IGF), or leptin. The BBB in humans expresses an insulin receptor, IR (Pardridge et al., 1985), a Tf receptor, TfR (Pardridge et al., 1987), an IGF receptor, IGFR (Duffy et al., 1988), and a leptin receptor, LEPR (Golden et al., 1997). *In vivo* studies involving intra-arterial infusion of the peptides show these BBB peptide receptors mediate the RMT of the peptide across the BBB, as demonstrated for insulin (Duffy and Pardridge, 1987), transferrin (Fishman et al., 1987; Skarlatos et al., 1995), IGF-1 and IGF-2 (Reinhardt and Bondy, 1994), and leptin (Kurrimbux et al., 2004). In addition to the endogenous peptide, these BBB receptors enable the RMT of certain peptidomimetic MAbs, which bind exofacial epitopes on the receptor, and this binding causes RMT of the MAb across the BBB in parallel with the BBB transport of the endogenous peptide. The RMT of a receptor-specific MAb was demonstrated for a MAb against the TfR, designated a TfRMAb (Pardridge et al., 1991), for a MAb against the human IR (HIR), designated a HIRMAb (Pardridge et al., 1995), and a MAb against the IGFR, designated the IGFRMAb (Shin et al., 2022; Yogi et al., 2022). Early work showed that biologics, which normally do not cross the BBB, were enabled to penetrate the BBB and induce *in vivo* CNS pharmacologic effects following intravenous (IV) administration providing the biologic was conjugated to a TfRMAb. Coupling of vasoactive intestinal peptide (VIP) to the murine OX26 MAb, which is specific for the rat TfR, via an avidin-biotin linker, caused as 65% increase in cerebral blood flow (CBF) following administration of the VIP-TfRMAb conjugate, whereas VIP alone had no effect on CBF (Bickel et al., 1993). Conjugation of nerve growth factor (NGF) to the OX26 TfRMAb, via a chemical linker, induced neuroprotection in an ocular transplant model (Friden et al., 1993).

The HIRMAb or TfRMAb undergoes RMT across the BBB without interference of the RMT of the endogenous ligand, insulin or Tf, because the MAb and the endogenous ligand have separate binding sites on different domains of the receptor. Insulin binds the HIR at that interface of the αCT and L1 domains (Menting et al., 2013), whereas the HIRMAb binds the first fibronectin domain (McKern et al., 2006). Tf binds the helical and protease-like domains of the TfR, but not the apical domain (Eckenroth et al., 2011), which is the site of binding of the TfRMAb. As the HIRMAb or TfRMAb undergoes RMT across the BBB, the antibody acts as a molecular Trojan horse to ferry into brain the attached biologic, which alone does not cross the BBB. In addition to the capillary endothelium of brain, neurons and glial cells behind the BBB also express the targeted receptors. Immuno-histochemistry of brain shows expression in neural cells of the IR (Pomytkin et al., 2018), the TfR (Moos et al., 1998), the IGFR (Garcia-Segura et al., 1997), and the LEPR (Mutze et al., 2006; Fujita and Yamashita, 2019). Therefore, the MAb that traverses the BBB via these RMT pathways may also deliver the fused therapeutic to the intracellular compartment of brain cells. This property of targeting the intracellular space in brain is illustrated in the case of brain delivery of an IgG-lysosomal enzyme fusion protein, which leads to the degradation of intracellular glycosaminoglycan aggregates in brain (Pardridge, 2022a).

The RMT of a MAb targeting an endogenous receptor on the BBB is a process of 3 sequential steps: (1) binding of the MAb to an exofacial epitope on the receptor (R) expressed on the luminal plasma membrane of the capillary endothelium followed by endocytosis of the MAb-R complex into the endothelium; (2) movement of the MAb-R complex through the intracellular compartment of the endothelial cell followed by release of the MAb from the receptor and recycling of the receptor back to the luminal membrane; and (3) exocytosis of the MAb from the abluminal membrane of the capillary endothelium into the interstitial space of brain. The kinetics of the brain uptake in the anesthetized Rhesus monkey of either a TfRMAb or a HIRMAb-lysosomal enzyme fusion protein was fit to a partly flow-partly compartmental mathematical model to evaluate the kinetics of these steps of the RMT process (Pardridge and Chou, 2021). The results of this model are summarized in Table 1, and these estimates of the rates of endocytosis, recycling, and exocytosis parallel the known kinetics of receptor-mediated endocytosis of the TfR or IR (Pardridge, 2021). There are important differences in the RMT pathway via the TfR as compared to the IR, owing to the much different plasma concentrations of the endogenous ligand. The plasma concentration of holo-Tf, 25,000 nM, is 5 log orders of magnitude greater than the plasma concentration of insulin (Table 1). As a consequence of the very high plasma concentration of holo-Tf, all of the TfR at the luminal membrane is in the form of the TfR-holo-Tf complex, whereas over 90% of the IR at the luminal membrane is in the form of the unoccupied IR.

**Table 1:** Kinetics of monoclonal antibody delivery across the blood–brain barrier via RMT on either the TfR or IR.

Transport component	TfR	IR
T_1/2_ of receptor endocytosis	5–10 min	30 min
T_1/2_ of MAb exocytosis	5 min	20 min
T_1/2_ of receptor recycling	20 min	20 min
T_1/2_ of receptor-MAb dissociation	3–30 min high affinity MAb <30 s for moderate or low affinity MAb	
Estimated k_on_ of MAb binding to receptor	10^6^ M^−1^ s^−1^	10^5^ M^−1^ s^−1^
Plasma concentration of endogenous ligand	Holo-Tf = 25,000 nM	Insulin = 0.3 nM
Total concentration of endothelial receptor	40 nM	24 nM
Concentration of luminal endothelial receptor	2 nM (TfR-MAb complex)	21 nM (unoccupied IR)

From Pardridge (2021). TfR, transferrin receptor; IR, insulin receptor; Tf, transferrin; T_1/2_, half-time; k_on_, association rate constant.

The engineering of MAb fusion proteins that target the RMT systems on the BBB was enabled following the cloning and sequencing of the variable regions of the heavy and light chains of the HIRMAb, or the TfRMAb, which was followed by the testing of the IgG fusion proteins in animal models of brain disease (Pardridge and Boado, 2012), and subsequently in clinical trials. The first clinical trial of the BBB Trojan horse technology tested the effects of a fusion protein of the chimeric HIRMAb, and L-α-iduronidase (IDUA), the lysosomal enzyme mutated in Mucopolysaccharidosis Type I (MPSI). The HIRMAb-IDUA fusion protein, designated valanafusp alfa, was administered to pediatric patients with MPSI for 52 weeks at 1–6 mg/kg/week (Giugliani et al., 2018). MPSI has severe effects in the CNS causing cognitive dysfunction and cerebral atrophy, and both aspects of this neurodegenerative disease were arrested after 1 year of treatment with the HIRMAb-IDUA fusion protein. A fusion protein of a MAb against the human TfR, and iduronate 2-sulfatase (IDS), the lysosomal enzyme mutated in MPSII, and designated pabinafusp alfa, was administered chronically by weekly IV infusion to pediatric patients with MPSII, and this treatment stabilized cognitive function and reduced CSF glycosaminoglycan, leading to the first market approval of a BBB-penetrating IgG-biologic fusion protein in Japan (Sonoda et al., 2022). The utilization of RMT pathways at the BBB, and the re-engineering of biologics as BBB-penetrating IgG-biologic fusion proteins, can be extended from orphan diseases, such as MPSI or MPSII, to neurodegenerative conditions, such as PD, as reviewed below.

The re-engineering of biologics for the treatment of PD assumes the RMT pathways at the BBB are intact in human PD. The data described below for animal models of PD show that the RMT of biologics is an active process in experimental PD. With regard to human PD, the BBB is intact based on brain scanning with positron emission tomography following the intravenous administration of ^82^Rb, a small molecule tracer (Fujita et al., 2021). In an MRI study with a small molecule contrast agent, the BBB is said to be leaky (Al-Bachari et al., 2020). However, the increases in BBB transfer coefficient for the contrast agent were minor, not detected in all regions of brain, and changes in PD were no different from the minor changes observed for cerebrovascular disease (Al-Bachari et al., 2020). The general intactness of the BBB in PD necessitated the disruption of the BBB by focused ultrasound to enable the brain delivery of a biologic, recombinant glucocerebrosidase, in PD (Meng et al., 2022).

## 2. Blood–brain barrier receptor-mediated transport of IgG-neurotrophin fusion proteins in Parkinson’s disease

### 2.1. Glial cell-derived neurotrophic factor

#### 2.1.1. GDNF delivery to brain

GDNF is a trophic factor for dopaminergic neurons (Lin et al., 1993), which made this neurotrophin a candidate for treatment of PD. Other neurotrophic factors may also be therapeutic in PD, but the problem with drug development of these agents is the lack of neurotrophin transport through the BBB (Bondarenko and Saarma, 2021). With respect to GDNF, this neurotrophin does not cross the BBB in the mouse (Kastin et al., 2003) or the primate (Boado and Pardridge, 2009). In the absence of BBB drug delivery technology, the PD drug developer must resort either to disrupting the BBB, or to a trans-cranial route of brain drug delivery via drug injection either into the CSF or via an intra-cerebral implant. GDNF delivery to brain following BBB disruption was tested with either the intra-carotid artery infusion of hypertonic solutions (Jiao et al., 1996) or focused ultrasound-microbubbles (Wang et al., 2012). BBB disruption is toxic to the brain (Pardridge, 2022b), and the BBB disruption approaches have not led to FDA approval of any drugs for brain disorders. Trans-cranial GDNF delivery to brain employs BBB avoidance strategies, such as intra-cerebroventricular (ICV) injection (Nutt et al., 2003) or intra-cerebral convection enhanced diffusion (CED; Lang et al., 2006). Both the ICV and the CED clinical trials of GDNF treatment in PD failed. The failure of the ICV route of GDNF delivery to brain was predictable based on the sponsor’s preclinical data, which showed that the injection of a neurotrophin into one lateral ventricle (LV) did not result in significant neurotrophin penetration into the brain (Yan et al., 1994). ICV drug delivery to brain results in drug distribution to the ependymal surface of the ipsilateral LV and the third ventricle, but negligible drug delivery to the contralateral LV or the parenchyma of brain. This is because CSF is rapidly exported from brain to blood via convection, whereas drug distribution from the ependymal surface of the ventricle into brain parenchyma occurs slowly via diffusion, which decreases logarithmically from the ependymal surface (Pardridge, 2022b). The failure of the GDNF CED clinical trial in PD can be traced to the minimal volume of brain that is exposed to drug with this delivery technology. GDNF was delivered to the primate brain with CED, and GDNF distribution in brain was measured by IHC and ELISA (Salvatore et al., 2006). The effective treatment volume following CED was 87–360 mm^3^, which is a small fraction of the volume of one putamen region of the human brain, 6,000 mm^3^ (Yin et al., 2009). The concentration of GDNF in brain was measured at various distances from the catheter tip, and the brain GDNF concentration decreased logarithmically (Salvatore et al., 2006), which is consistent with GDNF penetration into brain tissue via diffusion, not convection (Pardridge, 2022b). Given the history of the GDNF CED clinical trials in PD, there is concern that these failures will hinder future GDNF drug development for PD (Barker et al., 2020; Manfredsson et al., 2020). However, the efficacy of GDNF as a therapeutic in PD cannot be assessed from failed ICV or CED clinical trials, because these trans-cranial delivery approaches did not result in adequate GDNF delivery to the brain (Pardridge, 2022b). An alternative to the use of invasive BBB avoidance strategies is the re-engineering of GDNF to enable BBB transport of the neurotrophin via RMT across the BBB. This is possible by re-engineering GDNF as a fusion protein with either a HIRMAb or a TfRMAb, as discussed below. The trans-vascular route to brain is a preferred form of drug delivery to brain, because every neuron is perfused by its own blood vessel (Pardridge, 2002).

#### 2.1.2. HIRMAb-GDNF fusion protein

**Figure 1:**
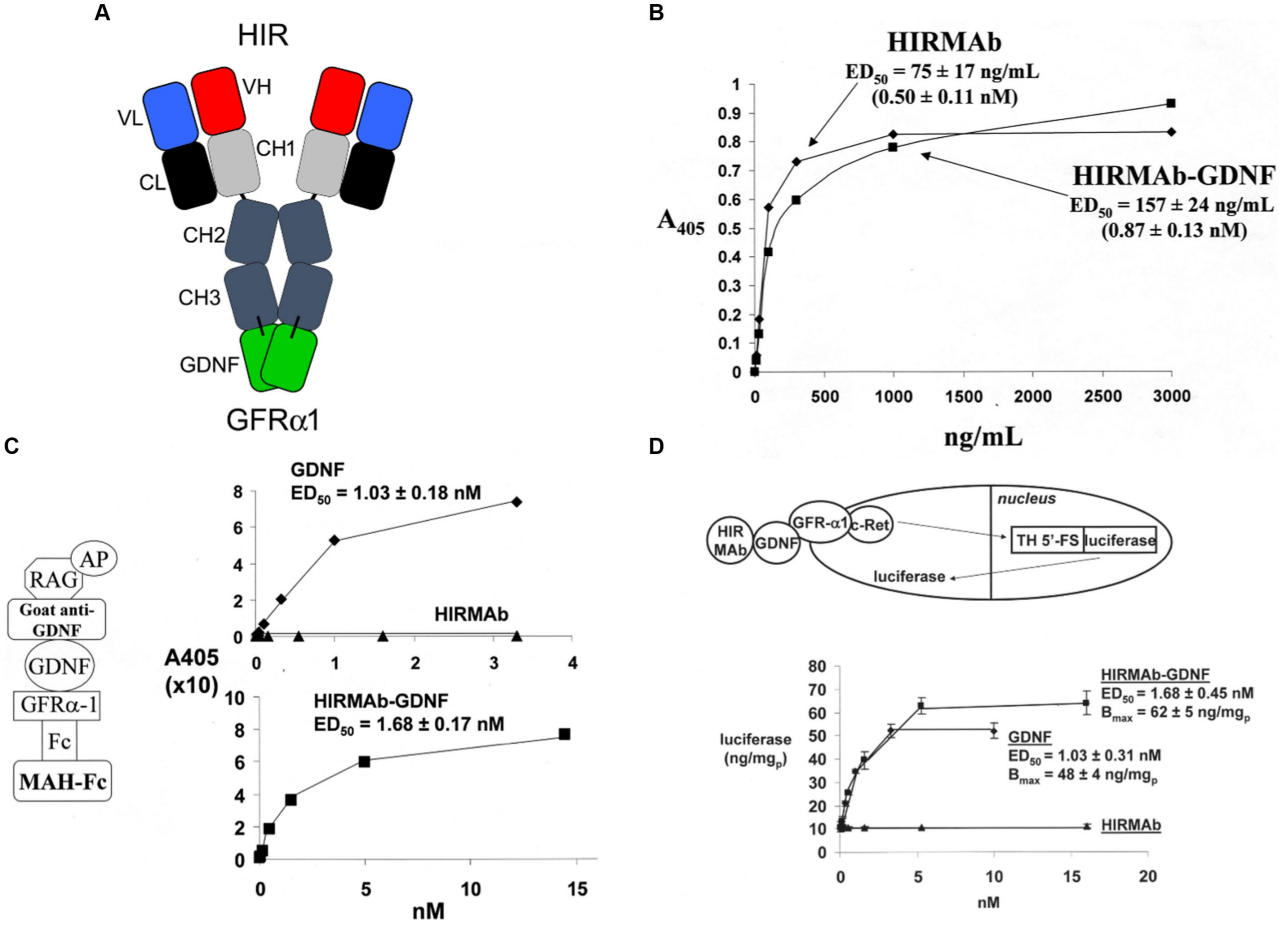
**(A)** Structure of HIRMAb-GDNF fusion protein where the mature human GDNF is fused to the carboxyl terminus of each heavy chain of the MAb directed against the human insulin receptor (HIR). The fusion protein binds 2 receptors: the HIR at the human or primate BBB to enable RMT across the BBB, and the GFRα1, to mediate GDNF action in brain. **(B)** The HIRMAb-GDNF fusion protein retains high affinity binding to the HIR in an ELISA using the HIR ECD as capture agent. The concentration of fusion protein that produces 50% of maximal binding, ED50, 0.87 ± 0.13 nM, is comparable to the ED50 of binding to the HIR of the original HIRMAb, 0.50 ± 0.11 nM. **(C)** The HIRMAb-GDNF fusion protein retains high affinity binding to the human GFRα1 in an ELISA assay. The GFRα1 ELISA design is shown on the left. The capture agent is a mouse anti-human (MAH)-Fc, which binds a GFRα1:Fc fusion, which binds either the GDNF domain of the IgG-GDNF fusion protein or recombinant human GDNF. The detector agent is a complex of a goat anti-GDNF antibody and a conjugate of alkaline phosphatase (AP) and rabbit anti-goat (RAG) secondary antibody. The concentration of HIRMAb-GDNF fusion protein that produces 50% of maximal binding, ED50, 1.68 ± 0.17 nM, is comparable to the ED50 of binding to the GFRα1 of human GDNF, 1.03 ± 0.18 nM. **(D)** The bio-activity of the HIRMAb-GDNF fusion protein, or GDNF, is assayed with human neural SK-N-MC cells that are permanently transfected with the c-ret kinase and a luciferase reporter plasmid under the influence of the 5′-flanking sequence (FS) of the rat tyrosine hydroxylase (TH) gene. The activation of the GFRα1/c-ret complex by a 24 h incubation of either GDNF alone, or the HIRMAb-GDNF fusion protein, is proportional to the luciferase enzyme activity in the cell lysate. The ED50 of luciferase gene expression activation by either GDNF or the HIRMAb-GDNF fusion protein is comparable to the ED50 values in the GFRα1 ELISA in panel **(C)**. The HIRMAb alone is not active in the bio-assay. Reprinted by permission from Boado et al. (2008).

GDNF was re-engineered for penetration of the human BBB by production of a HIRMAb-GDNF fusion protein. The mature human GDNF was fused to the carboxyl terminus of each heavy chain of the chimeric HIRMAb (Boado et al., 2008), and the structure of this fusion protein is shown in Figure 1A. The 134 amino acid sequence of the GDNF domain of the fusion protein is 100% aligned with amino acids 78–211 of the human preproGDNF (NP_000505). The design of the HIRMAb-GDNF fusion protein places the GDNF in a dimeric configuration, which replicates the natural dimeric structure of GDNF. A GDNF dimer binds a dimer of the GDNF receptor (GFR)α1 (Xu et al., 1998; Eketjall et al., 1999), and this hetero-tetrameric structure activates the c-ret kinase to mediate GDNF action (Cik et al., 2000; Parkash et al., 2008). The HIRMAb-GDNF fusion protein is bi-functional and binds both the insulin receptor, to enable RMT across the BBB, and the GDNF receptor, GFRα1. The high affinity binding of the HIRMAb-GDNF fusion protein to the HIR was comparable to the binding of the HIRMAb alone, as demonstrated by ELISA using the HIR ECD as the capture agent (Figure 1B). Retention of high affinity binding of the HIRMAb-GDNF fusion protein to the GFRα1 was shown by both ELISA (Figure 1C) and a bio-assay with human neural cells (Figure 1D). The design of the GFRα1 ELISA is shown on the left panel of Figure 1C. The concentration that causes 50% of maximal binding, ED50, in the GFRα1 ELISA of the HIRMAb-GDNF fusion protein was comparable to the ED50 of GDNF alone (Figure 1C, right panel). A GDNF bio-assay employed human SK-N-MC cells. This cell line expresses the GFRα1, but not the c-ret kinase (Hirata and Kiuchi, 2003), which mediates GDNF action following binding to the receptor. The SK-N-MC neural cell line was doubly transfected with c-ret kinase and a luciferase expression plasmid under the influence of the tyrosine hydroxylase (TH) promoter (Tanaka et al., 2003). Since GDNF increases TH gene expression (Xiao et al., 2002), extracellular GDNF results in increased intracellular luciferase gene expression in the transfected SK-N-MC cell line (Tanaka et al., 2003), as outlined in Figure 1D (top panel). Extracellular GDNF increased luciferase expression with an ED50 of 1.03 ± 0.03 nM, and the HIRMAb-GDNF fusion protein produced a comparable ED50 in this bio-assay of 1.68 ± 0.45 nM (Figure 1D, bottom panel). The GDNF trophic effects of the HIRMAb-GDNF fusion protein were also tested in an *in vivo* bio-assay using the middle cerebral artery occlusion (MCAO) model of stroke in rats. The HIRMAb domain of the HIRMAb-GDNF fusion protein binds the insulin receptor in humans and Old World primates, such as the Rhesus monkey (Pardridge et al., 1995), but not the insulin receptor in rodents (Zhou et al., 2012). Therefore, the neuroprotective activity of the HIRMAb-GDNF fusion protein in the MCAO model was examined after the intra-cerebral injection of 130 ug of the fusion protein (Boado et al., 2008). Since the fusion protein is 17% GDNF and 83% HIRMAb, this dose of the fusion protein is equal to 22 ug of GDNF, and is comparable to the dose of GDNF that is neuroprotective following intra-cerebral injection in the rat with experimental PD (Sullivan et al., 1998). The intra-cerebral injection of the HIRMAb-GDNF fusion protein caused a 77% reduction in hemispheric stroke volume in the MCAO model in rats (Boado et al., 2008). Chinese hamster ovary (CHO) cells were stably transfected with plasmid DNA encoding the HIRMAb-GDNF fusion protein, followed by propagation in a bioreactor in serum free medium, and the fusion protein was purified with affinity and ion exchange chromatography, followed by nanofiltration and diafiltration (Pardridge and Boado, 2009). A Good Laboratory Practice safety pharmacology and toxicology study was performed in 56 adult Rhesus monkeys with no adverse events observed at doses ranging from 2 to 50 mg/kg administered IV over a 60-h period. A GLP Tissue Cross-Reactivity study in 35 organs showed a comparable binding of the fusion protein to tissues from either humans or Rhesus monkeys (Pardridge and Boado, 2009).

The efficacy of the HIRMAb-GDNF fusion protein in experimental PD was tested in Rhesus monkeys, since the HIRMAb domain of the fusion protein cross-reacts with the insulin receptor in this primate (Pardridge et al., 1995). Experimental PD was produced in 6–12 kg Rhesus monkeys administered a single dose of 0.4 mg/kg of 1-methyl-4-phenyl-1,2,3,6-tetrahydropyridine (MPTP) infused in the carotid artery (Ohshima-Hosoyama et al., 2012). At 1 week after toxin infusion, the monkeys were treated with twice-weekly IV infusions of 1 or 5 mg/kg of HIRMAb-GDNF fusion protein, which was continued for an additional 11 weeks, but no neuroprotection was observed in this model (Ohshima-Hosoyama et al., 2012). This lack of neuroprotection in the primate PD model following HIRMAb-GDNF administration is attributed to the high dose of MTP, 0.4 mg/kg, used in the study. The dose of MPTP determines the size of the nigral-striatal lesion (Lama et al., 2021), and MPTP has a particular propensity to destroy nigral dopaminergic neurons in the primate, that is not generally observed in rodents (Duty and Jenner, 2011). A dose response study of intra-arterial MPTP in Rhesus monkeys was evaluated by comparison of the effects of 0, 0.07, 0.12, and 0.24 mg/kg MPTP (Tian et al., 2012). The 0.24 mg/kg dose of arterial MPTP causes a 100% reduction of striatal dopamine transporter (DAT), which is a pre-synaptic marker of loss of striatal nerve terminals (Tian et al., 2012). The 0.4 mg/kg dose of MPTP caused a 93% reduction in cell bodies in the substantia nigra immunoreactive for TH (Ohshima-Hosoyama et al., 2012). Therefore, the 0.4 mg/kg dose of intra-arterial MPTP caused a 93–100% ablation of dopaminergic neurons in the nigral-striatal tract. GDNF treatment is neuroprotective in PD only if there is present a sufficient number of viable neurons in the substantia nigra (Quintino et al., 2019). A BBB-penetrating IgG-GDNF fusion protein is neuroprotective in a model of experimental PD that produces a partial lesion of the nigra-striatal tract, as discussed in the next section. The study of Ohshima-Hosoyama et al. (2012) makes the claim that the low dose, but not the high dose, of the HIRMAb-GDNF fusion protein induces the formation of pre-malignant pancreatic neoplasms, which were designated as pancreatic intra-epithelial neoplasia (PanIn)-1. However, this claim is not valid, as PanIn-1 is not a pre-malignant lesion of the pancreas (Hruban et al., 2001), and PanIn nodules are found in 86% of human pancreases examined at autopsy (Longnecker and Suriawinata, 2022). Chronic administration of an IgG-GDNF fusion protein shows no evidence of toxicity in either primates (Pardridge and Boado, 2009), or in mice, as discussed in the next section.

#### 2.1.3. TfRMAb-GDNF fusion protein

**Figure 2:**
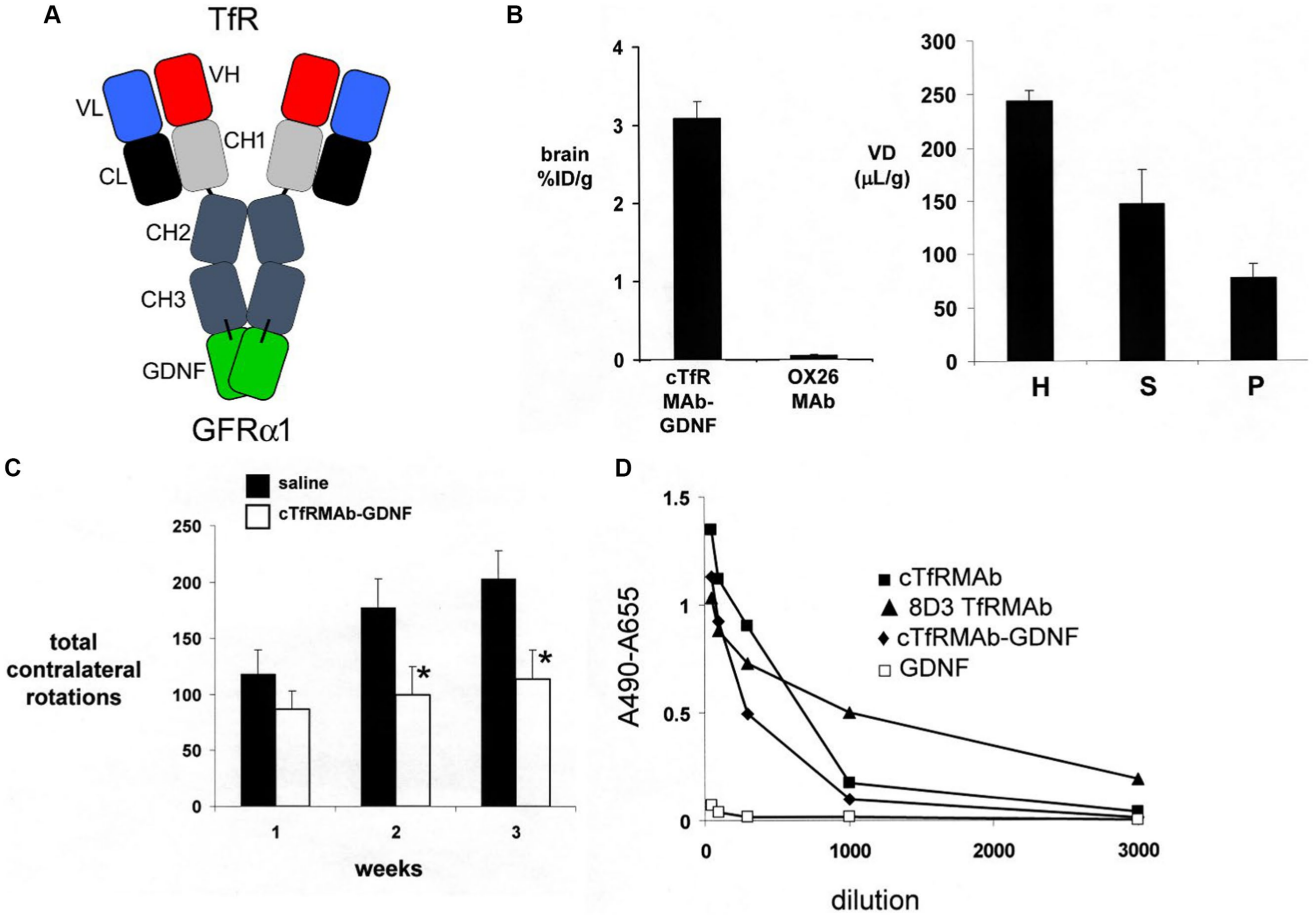
**(A)** Structure of TfRMAb-GDNF fusion protein where the mature human GDNF is fused to the carboxyl terminus of each heavy chain of the mouse/rat chimeric MAb against the mouse transferrin receptor (TfR), and designated the cTfRMAb-GDNF fusion protein. The fusion protein binds 2 receptors: the TfR at the mouse BBB to enable RMT across the BBB, and the GFRα1 to mediate GDNF action in brain. **(B)** Left panel: Brain uptake, measured as % of injected dose (ID) per gram brain, in the mouse of either the cTfRMAb-GDNF fusion protein, or the OX26 MAb against the rat TfR at 60 min after intravenous (IV) administration. The OX26 MAb does not recognize the mouse TfR and does not enter mouse brain. Right panel: Capillary depletion analysis shows the volume of distribution (VD) of the cTfRMAb-GDNF fusion protein in the homogenate (H), post-vascular supernatant (S), and vascular pellet (P) in mouse brain at 60 min after IV administration. Panels **(A,B)** reprinted with permission from Zhou et al. (2010a). **(C)** *In vivo* bioactivity of the TfRMAb-GDNF fusion protein in mice with experimental PD induced by the injection of 6 ug of 6-hydroxydopamine in each of 2 locations of the right striatum. Mice were treated intravenously either with saline or with the TfRMAb-GDNF fusion protein, 1 mg/kg, administered every 2 days for 3 weeks, starting 1 h after toxin injection. Apomorphine-induced rotation behavior was measured weekly. Data are mean ± SE (*n* = 9 mice/group). Statistical differences from the saline treated animals at 2 and 3 weeks are *p* < 0.05 (*). Reprinted with permission from Fu et al. (2010). **(D)** Anti-drug antibody (ADA) response in mice after 12 weeks of treatment with the TfRMAb-GDNF fusion protein, 2 mg/kg IV, given twice-weekly. The terminal serum from the treated mice were pooled and tested at dilutions ranging from 1:50 to 1:3000 against 4 different capture agents in the ADA ELISA: the chimeric cTfRMAb against the mouse TfR, the hybridoma generated rat 8D3 MAb against the mouse TfR, the cTfRMAb-GDNF fusion protein, or human GDNF. The data show the low titer ADA response is directed against the TfRMAb domain of the fusion protein, with no response against the GDNF domain. Reprinted from Zhou et al. (2011a).

A partial lesion of the nigra-striatal tract in mouse models of experimental PD is produced following the intra-cerebral injection of 6-hydroxydopamine in the striatum (Tieu, 2011; Jagmag et al., 2015). Since the HIRMAb domain of the HIRMAb-GDNF fusion protein does not recognize the murine insulin receptor (Zhou et al., 2012), mice with experimental PD were treated with an IgG-GDNF fusion protein that is active in the mouse. The rat/mouse chimeric form of the rat 8D3 MAb against the mouse TfR, designated cTfRMAb, had been engineered (Pardridge and Boado, 2012), which enabled engineering of a cTfRMAb-GDNF fusion protein (Zhou et al., 2010a). The structure of the cTfRMAb-GDNF fusion protein is shown in Figure 2A, which places the GDNF domain of the fusion protein in a dimeric configuration. The brain uptake of the cTfRMAb-GDNF fusion protein in the mouse is high 3.1 ± 0.2% ID/gram at an ID of 1 mg/kg (Figure 2B, left panel). In contrast the brain uptake of the OX26 MAb against the rat TfR, which does not recognize the mouse TfR (Lee et al., 2000), is at the background level reflecting entrapment of the OX26 MAb in the blood volume of the brain in the mouse (Figure 2A, left panel). The brain volume of distribution (VD) of the cTfRMAb-GDNF fusion protein in a homogenate of brain is high, 244 ± 19 uL/gram, which is 23-fold higher than the brain plasma volume in the mouse, which is 11 uL/gram (Lee et al., 2000). Capillary depletion analysis shows the VD in the post-vascular supernatant is 60% of the VD in the total homogenate (Figure 2B, right panel), which indicates that 60% of the cTfRMAb-GDNF fusion protein bound by the BBB is transcytosed within 60 min after IV administration (Zhou et al., 2010a). The bi-functionality of the cTfRMAb-GDNF fusion protein was demonstrated by showing the affinity of binding of the fusion protein to the mouse TfR was comparable to the affinity of the cTfRMAb, and the binding of the fusion protein to the human GFRα1 was comparable to GDNF alone in either the human GFRα1 ELISA or the human SK-N-MC bio-assay (Zhou et al., 2010a). Human GDNF is active at the mouse GFRα1, owing to the high amino acid identity, 93%, of mouse (NP_034405) versus human (P39905) mature GDNF, and mouse (P97785) versus human (NP_665736) GFRα1 ECD.

Experimental PD was produced in adult male C57BL/6 mice following the intra-cerebral injection of 6 ug of 6-hydroxydopamine in each of 2 locations of the right striatum (Fu et al., 2010). At 1 h after toxin administration, mice were treated every other day with IV injections of either saline or 1 mg/kg cTfRMAb-GDNF fusion protein for 3 weeks prior to euthanasia. Mice treated with the cTfRMAb-GDNF fusion protein showed a 44% reduction in apomorphine-induced rotation behavior at 2 and 3 weeks (Figure 2C). The fusion protein treated mice exhibited a 62 and 45% reduction in amphetamine-induced rotation behavior at 2 and 3 weeks (Fu et al., 2010). The vibrissae-elicited forelimb placing test is a measure of abnormal motor activity in mice with experimental PD (Anstrom et al., 2007). The mice treated with the cTfRMAb-GDNF fusion protein showed a 121% increase in placing score on the lesioned side (Fu et al., 2010). TH enzyme activity in homogenates of striatum and frontal cortex was measured with [3,5-^3^H]-L-tyrosine. The 6-hydroxydopamine lesion produced a 79% reduction in TH enzyme activity in the striatum of the lesioned side, and treatment with the cTfRMAb-GDNF fusion protein caused a 272% increase in striatal TH enzyme activity (Fu et al., 2010). The TH enzyme activity in the frontal cortex was 3.5% of the TH enzyme activity in the striatum, and treatment with the cTfRMAb-GDNF fusion protein caused no change in TH enzyme activity in the cortex (Fu et al., 2010). The low level of TH enzyme activity in the cortex is produced by the presence of intra-cortical interneurons (Benavides-Piccione and DeFelipe, 2007), which migrate to the cortex (Wonders and Anderson, 2006).

The potential toxicity of chronic treatment of mice with the cTfRMAb-GDNF fusion protein was examined in 3 month old C57BL/6 mice, which were treated twice-weekly for 12 weeks with either saline or 2 mg/kg/dose of the cTfRMAb-GDNF fusion protein (Zhou et al., 2011a). The treatment groups included 12 male and 12 female mice. The cTfRMAb-GDNF fusion protein was radiolabeled with [^3^H]-N-succinimidyl propionate, and the [^3^H]-cTfRMAb-GDNF fusion protein was injected IV at the start and at the end of the 12-week treatment study for a brain uptake and pharmacokinetics (PK) analysis. During the course of the study there were no injection reactions and no change in body weight between the saline and cTfRMAb-GDNF fusion protein treatment groups. Organ histology by hematoxylin and eosin staining was performed on brain, kidney, liver, spleen, heart, and pancreas. The tissue histology was examined by a certified neuropathologist or gastro-intestinal pathologist, which showed no change in tissue histology for brain, pancreas, or other organs. There was no change in 23 tests of serum chemistry, including no change in serum iron or Tf. The level of brain uptake, the BBB permeability-surface area (PS) product, which is a measure of transport of the fusion protein via the BBB TfR, and the plasma clearance rate, which is a measure of peripheral TfR, were unchanged at the end of the study as compared to the start of the study. The unchanged BBB PS product for the cTfRMAb-GDNF fusion protein shows there is no down- or up-regulation of the TfR at the BBB following chronic treatment. The unchanged plasma clearance of the fusion protein shows there is no down- or up-regulation of the TfR in peripheral organs following chronic treatment with the cTfRMAb-GDNF fusion protein. The potential for immune reactions against the fusion protein was examined with an anti-drug antibody (ADA) ELISA, which measured ADA titers in serum taken at the end of the study. Following chronic treatment of mice with the cTfRMAb-GDNF fusion protein, the ADA titer was <1 OD/uL serum, where OD = optical density (Zhou et al., 2011a). ADA titers <20 OD/uL are considered evidence for immune tolerance to biologics (Dickson et al., 2008). The capture agent of the ADA ELISA was varied and included either the cTfRMAb-GDNF fusion protein, GDNF alone, the cTfRMAb alone, or the original rat 8D3 antibody, and the ADA titers for serial dilutions of pooled treatment serum are shown in Figure 2D. This analysis shows the low titer immune response generated in mice treated chronically with the cTfRMAb-GDNF fusion protein is directed against the cTfRMAb domain, and not the GDNF domain (Zhou et al., 2011a). This low titer ADA response against the cTfRMAb domain of the fusion protein did not affect RMT delivery of the fusion protein across the BBB, because the brain uptake of the fusion protein at the end of the treatment study was unchanged relative to the brain uptake at the start of the chronic treatment study (Zhou et al., 2011a).

In summary, re-engineering GDNF as a BBB-penetrating IgG-GDNF fusion protein enables neuroprotection in experimental PD with chronic systemic administration of 1 mg/kg of the fusion protein, and this dose has no effect on tissue histology, serum chemistry, body weight and causes no significant ADA immune response. Re-engineering human GDNF as an IgG-GDNF fusion protein that penetrates the BBB via RMT can enable the future development of GDNF therapeutics for PD that are delivered to brain following non-invasive systemic administration.

### 2.2. Erythropoietin

#### 2.2.1. Erythropoietin and Parkinson’s disease

Erythropoietin (EPO) is a potential neurotrophic factor treatment in PD and other neurodegenerative conditions (Rey et al., 2019). The intra-cerebral injection of EPO has both anti-oxidant and anti-apoptotic activity in the 6-hydroxydopamine model of experimental PD in the mouse (Thompson et al., 2020). The EPO receptor (EPOR) is expressed in dopaminergic neurons in the substantia nigra (Marcuzzi et al., 2016). Early work showed that the intra-cerebral injection of EPO was neuroprotective in a MPTP mouse model (Genc et al., 2001), a 6-hydroxydopamine mouse model (Signore et al., 2006), and a 6-hydroxydopamine rat model (Xue et al., 2007) of experimental PD. The neuroprotective action of EPO in experimental PD observed following the direct intra-cerebral injection of the neurotrophin could not be replicated following the systemic administration of daily intra-peritoneal (IP) injections of 5,000 units of EPO/kg (Xue et al., 2007). As discussed in the next section, EPO does not cross the intact BBB. Therefore, peripheral administration of EPO is neuroprotective only if there is BBB disruption (Catania et al., 2002). The BBB is intact in human PD even to small molecule imaging agents (Fujita et al., 2021). Since the BBB is intact in human PD, a neuroprotective effect from EPO would not be expected in PD following systemic administration of the neurotrophin. In a failed clinical trial of systemic EPO in PD, patients were treated by IV infusion of 40,000 IU of EPO, which is about 500 IU/kg; the EPO was administered twice-weekly for 5 weeks, but this treatment had no effect on the Unified Parkinson’s Disease Rating Scale (UPDRS)-III (Jang et al., 2014). This dose of EPO is higher than the EPO dose, about 150 IU/kg, used to increase hematocrit in chronic renal disease (Thadhani et al., 2018), and a longer duration of treatment of PD subjects with EPO would be expected to cause a prohibitive increase in blood hematocrit leading to polycythemia. In summary, there are 2 problems limiting the treatment of PD with systemic EPO. First, EPO does not cross the BBB. Second, systemic EPO would have prohibitive effects on hematopoiesis. Solutions have been proposed for both of these limiting factors in the drug development of EPO for PD as reviewed below.

#### 2.2.2. Erythropoietin and the blood–brain barrier

The volume of distribution (VD) of EPO in the primate brain is no different from the brain plasma volume (Vo) (Boado et al., 2010a). Similarly, the primate brain VD of GDNF is no different from the Vo (Boado and Pardridge, 2009). When the VD of a drug equals the Vo in brain, there is no transport of the neurotrophin across the BBB, as the molecule is confined to the plasma volume of brain. However, it is frequently proposed that EPO crosses the BBB (Rey et al., 2019). The evidence cited for the BBB transport of EPO is the finding that the concentration of EPO in CSF increases following systemic administration (Ehrenreich et al., 2002). However, drug entry into CSF is a measure of drug passage across the blood-CSF barrier formed by the choroid plexus lining the walls of the ventricles, and is not a measure of drug transport across the BBB at the brain capillary endothelium. Owing to the relative leakiness of the choroid plexus, all molecules in blood enter CSF at a rate inversely related to molecular weight (Pardridge, 2022b). In contrast to CSF, the concentration of EPO in parenchyma of non-injured brain is not increased following systemic administration of EPO and saline clearance of the plasma volume of brain (Lieutaud et al., 2008). EPO does not enter brain from plasma in the absence of BBB disruption (Catania et al., 2002). Other evidence used to support the hypothesis of BBB transport of EPO is the finding of neuroprotection following systemic administration of EPO in traumatic brain injury (TBI; Brines et al., 2000). However, the BBB is disrupted soon after TBI (Fukuda et al., 1995), which is the mechanism of EPO entry into brain in TBI (Lieutaud et al., 2008). It has been proposed that the EPO receptor (EPOR) is expressed at the BBB on the basis of an electron microscopic study (Brines et al., 2000); however, inspection of these electron micrographs shows the microvascular immunoreactive EPOR is found exclusively on the abluminal side of the brain microvasculature. RMT of EPO across the BBB via the EPOR would require expression of the receptor on the luminal membrane of the brain endothelium (Pardridge, 2022b). Since EPO does not cross the intact BBB, it is necessary to re-engineer this neurotrophin as an IgG-EPO fusion protein that gains access to brain via RMT across the BBB, as reviewed in sections 2.2.4 and 2.2.5.

#### 2.2.3. Erythropoiesis and EPO pharmaceuticals for brain

A limiting factor in the development of EPO biologics for brain is the hematopoietic effects caused by systemic administration of EPO. The goal is to develop a formulation of EPO that crosses the BBB to induced neuroprotection in PD, or other neurodegenerative diseases, without enhancing erythropoiesis or raising the blood hematocrit. The hematopoietic effect of EPO is directly proportional to the plasma area under the concentration curve (AUC; Elliott et al., 2008). EPO has a relatively prolonged blood residence time, which leads to a high plasma AUC. However, removal of sialic residues from the carbohydrate domain of a glycoprotein, such as EPO, triggers rapid removal of the asialo-glycoprotein from plasma via uptake into liver mediated by a hepatic asialoglycoprotein receptor. Similarly, the removal of sialic acid groups from EPO results in rapid removal from plasma of the asialoEPO in rats following IV administration (Erbayraktar et al., 2003). The half-time (T_1/2_) of EPO in plasma is reduced >200-fold following desialation; the plasma T_1/2_ of EPO and asialoEPO is 5.6 h and 1.4 min, respectively. Predictably, EPO treatment of rats increases blood hemoglobin 25%, whereas treatment with asialoEPO has no effect on blood hemoglobin (Erbayraktar et al., 2003). However, the use of asialoEPO as a neuroprotective agent is still problematic, because asialoEPO does not cross the BBB. Nevertheless, asialoEPO, also called NeuroEPO, has been administered to patients with PD via weekly trans-nasal delivery of 1 mL solutions containing 1 mg of NeuroEPO (Bringas Vega et al., 2022). The trans-nasal route of drug delivery results in drug distribution to blood, not brain (Pardridge, 2022b), and asialoEPO that moves from the nose to plasma will still be cleared with a T_1/2_ of <5 min. An alternative formulation of EPO that is designed to not have a hematopoietic effect is carbamylated EPO, or CEPO (Leist et al., 2004). Receptor ligand carbamylation structurally alters lysine residues, which results in loss of ligand affinity for the target receptor (Weisgraber et al., 1978). Carbamylation of EPO causes a complete loss of CEPO binding to the EPOR, and CEPO has no hematopoietic effect (Leist et al., 2004). However, CEPO was proposed as a neuroprotective agent based on the hypothesis of a unique EPOR in brain that still bound CEPO (Leist et al., 2004). The hypothesis of a second EPOR specific for the CNS is not consistent with the observation that there is only a single EPOR, which forms a homo-dimer and that the neuroprotective effects of EPO are mediated via this classical EPOR homo-dimer (Um et al., 2007). The idea of a neural-specific EPOR, which is reactive with CEPO, is still hypothesized to exist as a hetero-dimeric structure of one EPOR subunit and one subunit of CD131 common beta subunit (Lee et al., 2021). The problem with the hypothesis that CD131 acts as a co-receptor with monomeric EPOR is that biophysical studies show there is no interaction between CD131 and the EPOR (Cheung Tung Shing et al., 2018). Setting aside the issue as to whether there is a neuroprotective-specific receptor for EPO, the development of EPO analogues, such as NeuroEPO or CEPO, which have no hematopoietic effect, still do not cross the BBB. These dual problems of lack of EPO transport across the BBB, and the requirement for an EPO formulation with minimal hematopoietic effect, can be solved by re-engineering EPO as an IgG-EPO fusion protein, where the IgG domain triggers IgG-EPO entry into brain from blood via RMT across the BBB, as discussed in Sections 2.2.4 and 2.2.5.

#### 2.2.4. HIRMAb-EPO fusion protein

**Figure 3:**
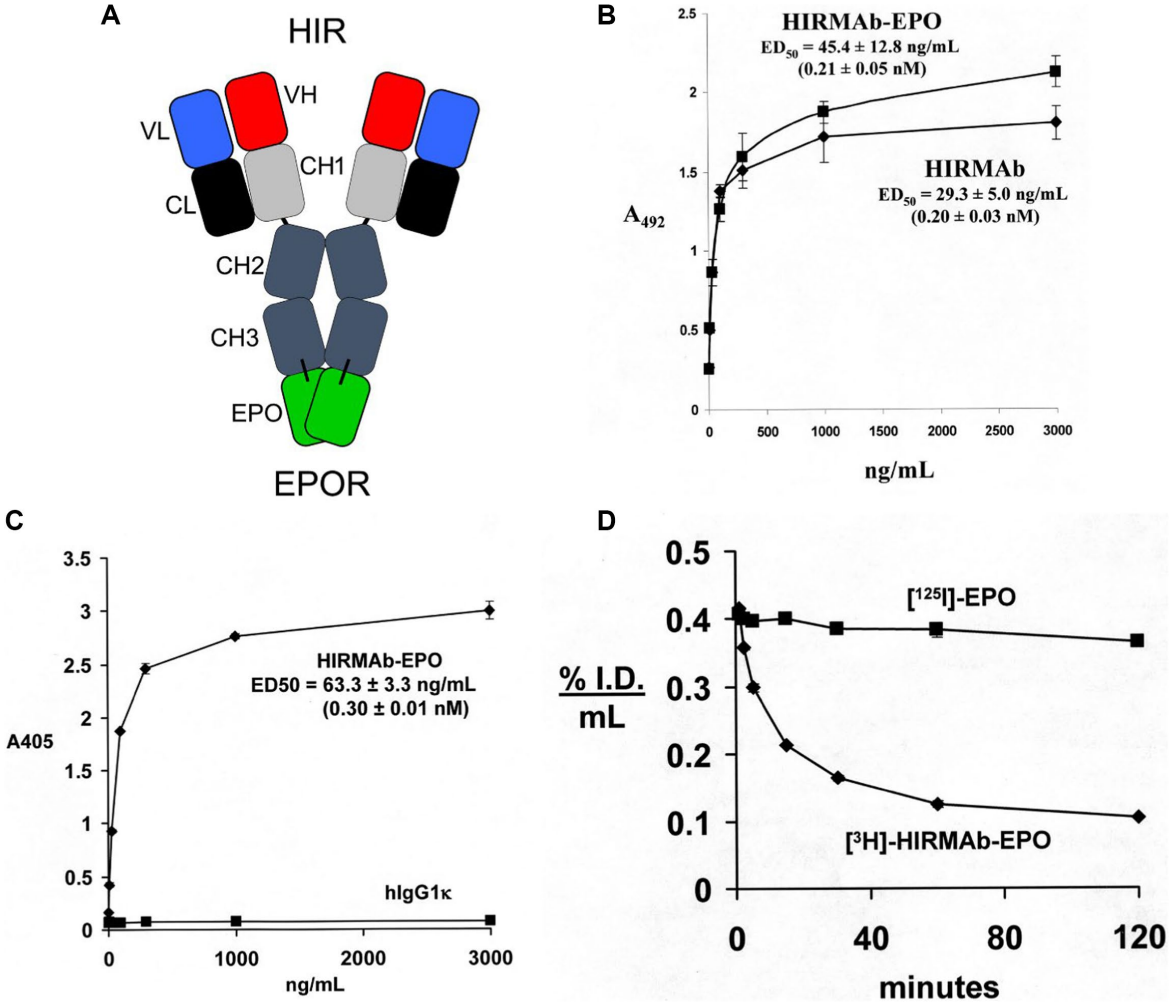
**(A)** Structure of HIRMAb-erythropoietin (EPO) fusion protein where the mature human EPO is fused to the carboxyl terminus of each heavy chain of the MAb directed against the human insulin receptor (HIR). The fusion protein binds 2 receptors: the HIR at the human or primate BBB to enable RMT across the BBB, and the EPO receptor (EPOR) to mediate EPO action in brain. **(B)** The HIRMAb-EPO fusion protein retains high affinity binding to the HIR in an ELISA assay. The concentration of fusion protein that produces 50% of maximal binding, ED50, 0.21 ± 0.05 nM, is comparable to the ED50 of binding to the HIR of the original HIRMAb, 0.20 ± 0.03 nM. **(C)** The HIRMAb-EPO fusion protein retains high affinity binding to the human EPOR in an ELISA assay. The concentration of fusion protein that produces 50% of maximal binding, ED50, 0.30 ± 0.01 nM, is comparable to the KD of binding to the EPOR of the human EPO in a radio-receptor assay (Boado et al., 2010a). There is no binding to the EPOR by the human IgG1k isotype control antibody. **(D)** Time profile of plasma concentration, measured as % injected dose (ID)/mL, of either [^125^I]-EPO or [^3^H]-HIRMAb-EPO fusion protein in the Rhesus monkey after IV administration. Panels **(A–D)** reprinted with permission from Boado et al. (2010a).

EPO was re-engineered for penetration of the human BBB by production of a HIRMAb-EPO fusion protein. The mature human EPO was fused to the carboxyl terminus of each heavy chain of the chimeric HIRMAb (Boado et al., 2010a), and the structure of this fusion protein is shown in Figure 3A. The 166 amino acid sequence of the EPO domain of the fusion protein is 100% aligned with amino acids 28–193 of the human EPO precursor (NP_000790). This design places the EPO is a dimeric configuration. The EPO monomer binds a dimer of the EPO receptor (EPOR; Syed et al., 1998). However, an EPO dimer is more active than an EPO monomer (Sytkowski et al., 1998). The high affinity binding of the HIRMAb-EPO fusion protein to the HIR is comparable to the binding of the HIRMAb alone (Figure 3B). High affinity binding of the HIRMAb-EPO fusion protein to the human EPOR ECD is also observed, with an ED50 of 0.30 ± 0.01 nM (Figure 3C), which was comparable to the KD of EPO binding to the EPOR ECD in a radio-receptor assay, KD = 0.17 ± 0.09 nM (Boado et al., 2010a). The biological activity of the EPO domain of the HIRMAb-EPO fusion protein was confirmed with human TF-1 cells (Kitamura et al., 1989). Addition to these cells of the HIRMAb-EPO fusion protein caused a dose-dependent increase in thymidine incorporation with an ED50 of 0.10 nM (Boado et al., 2010a). The brain uptake, and plasma pharmacokinetics (PK) of EPO and the HIRMAb-EPO fusion protein were measured in the adult Rhesus monkey following radio-labeling of each protein. Human EPO was radio-labeled with the [^125^I]-Bolton-Hunter reagent, and the HIRMAb-EPO fusion protein was radio-labeled with [^3^H]-N-succinimidyl propionate (Boado et al., 2010a). The plasma concentration of each protein was measured over 120 min after IV administration, as shown in Figure 3D. A PK analysis showed the plasma AUC is 13-fold lower for HIRMAb-EPO fusion protein as compared to EPO. The brain VD of EPO, 9 ± 1 uL/gram, in the primate is no different from the brain plasma volume, which indicates EPO does not cross the BBB (Boado et al., 2010a). In contrast, the brain VD of the HIRMAb-EPO fusion protein was 260 ± 11 uL/gram, and capillary depletion analysis showed the majority of the fusion protein in brain at 2 h after administration was in the post-vascular volume of brain, indicating the fusion protein had fully transcytosed through the BBB (Boado et al., 2010a). Re-engineering of EPO as the HIRMAb-EPO fusion protein fulfills both criteria for development of EPO as a biologic for brain disease: (a) penetration of the BBB, and (b) large reduction in the plasma AUC of EPO, which causes a proportionate reduction in erythropoietic action of the EPO. So as to test both the neuroprotective and hematopoietic activity following chronic administration of a BBB-penetrating IgG-EPO fusion, mice with experimental PD were treated with a mouse-specific TfRMAb-EPO fusion protein, where the TfRMAb domain is a rat/mouse chimeric antibody derived from the 8D3 antibody against the mouse TfR, as described in the next section.

#### 2.2.5. TfRMAb-EPO fusion protein

**Figure 4:**
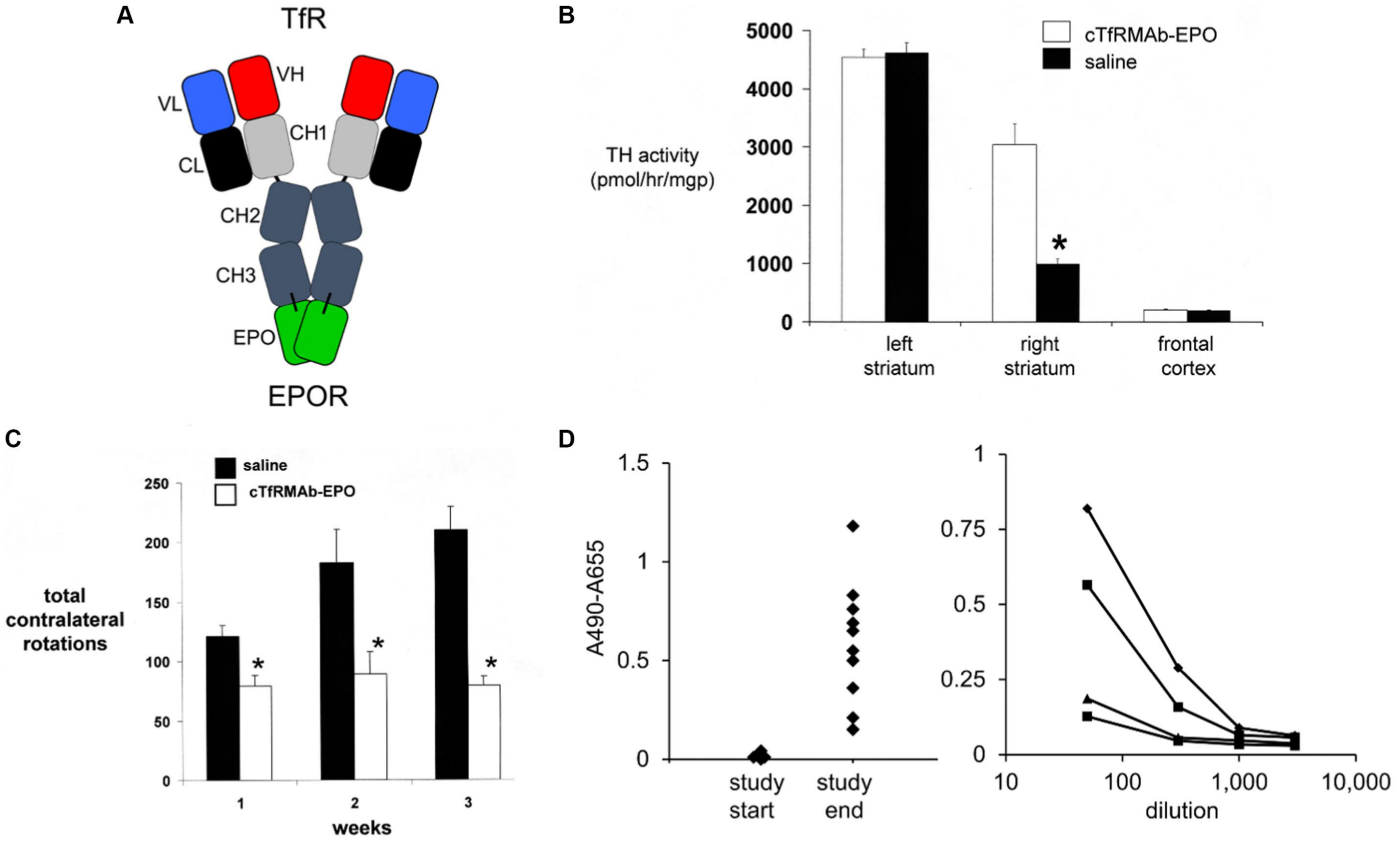
**(A)** Structure of TfRMAb-EPO fusion protein where the mature human EPO is fused to the carboxyl terminus of each heavy chain of mouse/rat chimeric MAb against the mouse transferrin receptor (TfR), and designated the cTfRMAb-EPO fusion protein. The fusion protein binds 2 receptors: the TfR at the mouse BBB to enable RMT across the BBB, and the EPOR to mediate EPO action in brain. Reprinted with permission from Zhou et al. (2010b). **(B)** Brain tyrosine hydroxylase (TH) enzyme activity in the left striatum (non-lesioned side), the right striatum (lesioned side), and the frontal cortex of mice with experimental PD following 3 weeks of treatment with either saline (closed bars) or 1 mg/kg cTfRMAb-EPO fusion protein (open bars) given every 2 days starting 1 h after 6-hydroxydopamine injection in 2 areas of the right striatum. Statistical differences from the saline-treated animals are *p* < 0.001 (*). **(C)** Apomorphine-induced rotation behavior at weekly intervals after toxin administration over the course of the 3-week treatment period. Statistical differences from the saline-treated animals are *p* < 0.005 at weeks 1 and 2 and *p* < 0.001 at week 3 (*). **(D)** Left panel: Anti-drug antibody (ADA) response at a plasma dilution of 1:50 in each mouse with experimental PD treated with the cTfRMAb-EPO fusion protein at the start and end of the 3-week treatment period. Right panel: Absorbance at dilutions (1:50, 1:300, 1:1000, and 1:3000) of plasma from four mice in the cTfRMAb-EPO fusion protein treatment group, including two mice that reacted the highest and two mice that reacted the lowest, in the screen at 1:50 dilution (left panel). Panels **(B–D)** from Zhou et al. (2011b).

To enable testing of a BBB-penetrating IgG-EPO fusion protein in a mouse model of experimental PD, human EPO was re-engineered as a TfRMAb-EPO fusion protein (Zhou et al., 2010b), as outlined in Figure 4A. The mature human EPO was fused to the carboxyl termini of both heavy chains of the rat/mouse chimeric TfRMAb derived from the variable regions of the rat 8D3 MAb against the mouse TfR, and from the constant regions of mouse IgG1κ. The TfRMAb-EPO fusion protein bound to the mouse EPOR ECD with high affinity, EC50 = 0.33 ± 0.04 nM (Zhou et al., 2010b), which is consistent with the 82% amino acid identity between the mature murine EPO (NP_031968) and human EPO (NP_000790). The plasma clearance of the TfRMAb-EPO fusion protein in the mouse, 5.9 ± 0.3 mL/min/kg at an injection dose of 0.1 mg/kg (Zhou et al., 2010b), is 14-fold faster than the plasma clearance of EPO in the mouse, 0.41 ± 0.03 mL/min/kg (Kato et al., 1998). This log order increase in plasma clearance of EPO following fusion to the TfRMAb is predicted to greatly reduce the hematopoietic effect of the EPO domain of the fusion protein, as described below. The brain uptake of the TfRMAb-EPO fusion protein in the mouse was high, 2.0 ± 0.1 %ID/gram (Zhou et al., 2010b), compared to the mouse brain uptake, 0.06 ± 0.01 %ID/gram, of the OX26 antibody, which does not recognize the mouse TfR, and does not cross the BBB in the mouse (Lee et al., 2000). Capillary depletion analysis showed the TfRMAb-EPO fusion protein rapidly transcytosed through the BBB to reach the post-vascular space in brain within 60 min of IV administration (Zhou et al., 2010b).

The neuroprotective effect of the TfRMAb-EPO fusion protein in experimental PD was tested in the mouse following the intra-cerebral injection of 6 ug of 6-hydroxydopamine in each of 2 regions of the right striatum (Zhou et al., 2011b). Mice were treated by IV injection of either saline or 1 mg/kg of the TfRMAb-EPO fusion protein given every 2 days for 3 weeks starting 1 h after toxin injection. This model produced a 78% reduction in striatal TH enzyme activity on the lesioned side (Figure 4B). Treatment with the TfRMAb-EPO fusion protein caused a 306% increase in striatal TH enzyme activity compared to the saline control (Figure 4B). Fusion protein treatment had no effect on the low level of TH activity in the frontal cortex (Figure 4B). The fusion protein-mediated increase in striatal TH activity correlated with an improvement in apomorphine-induced motor behavior, as drug induced rotation was reduced 35, 51, and 62% at 1, 2, and 3 weeks after toxin administration (Figure 4C). Similarly, amphetamine-induced rotations were reduced 62 and 65% at 2 and 3 weeks after toxin administration in the fusion protein treated mice (Zhou et al., 2011b). The vibrissae-elicited forelimb placing test score was reduced 80% in the saline treated PD mice, and this score was increased 132% by fusion protein treatment (Zhou et al., 2011b).

The hematopoietic effect of chronic treatment of mice with the TfRMAb-EPO fusion protein was assessed by measurement of hematocrit (Hct; Zhou et al., 2011b). A dose of 1 mg/kg of the TfRMAb-EPO fusion protein is equivalent to a dose of 0.2 mg/kg of EPO, since the EPO domain constitutes 20% of the fusion protein, based on amino acid sequence of the TfRMAb and EPO domains of the fusion protein. A dose of 0.2 mg/kg of EPO is equivalent to a dose of 20,000 units/kg, since 1 unit = 10 ng of EPO. The Hct increased from 48 ± 1% at the start of the study to 53 ± 2% and 54 ± 2% at 2 and 3 weeks of TfRMAb-EPO fusion protein chronic treatment at an EPO equivalent dose of 20,000 units/kg (Zhou et al., 2011b). In contrast, the Hct increased from 48 ± 1% at the start of treatment to 73 ± 2% and 84 ± 4% after 2 and 4 weeks of subcutaneous administration of EPO in mice every other day (Grignaschi et al., 2007). The dose of EPO, 4,000 units/kg, that produced these large increases in Hct (Grignaschi et al., 2007), is 5-fold lower than the equivalent dose of EPO administered via the cTfRMAb-EPO fusion protein (Zhou et al., 2011b). Fusion of EPO to the TfRMAb both enables neuroprotection following systemic administration of the EPO fusion protein, and abrogates the hematopoietic effect of systemic EPO treatment.

The immune response generated by chronic dosing of mice with the cTfRMAb-EPO fusion protein was determined with an ADA ELISA (Zhou et al., 2011b). The optical density (OD) at a 1:50 dilution of 100 μL of terminal serum taken from the PD mice treated with the TfRMAb-EPO fusion protein was measured at the start and end of the 3-week treatment study (Figure 4D, left panel). Serial dilutions were measured for 4 mice (the 2 mice with the highest ADA titer and the 2 mice with the lowest ADA titer), and all mice had no measurable ADA titer at a dilution of 1,000 (Figure 4D, right panel). The average OD/uL undiluted serum was 0.3. This is a very low ADA titer, as immune tolerance is indicated by an ADA titer <20 OD/uL (Dickson et al., 2008).

In summary, re-engineering EPO as a BBB-penetrating IgG-EPO fusion protein enables neuroprotection in experimental PD with systemic administration of 1 mg/kg, and this dose causes only a minor increase in Hct, and no significant immune response. Re-engineering human EPO as an IgG-EPO fusion protein that penetrates the BBB via RMT can enable the future development of EPO therapeutics for PD that can be delivered to brain following non-invasive systemic administration.

## 3. Blood–brain barrier receptor-mediated transport of IgG-decoy receptor fusion protein in Parkinson’s disease

### 3.1. TNF-alpha and Parkinson’s disease

Tumor necrosis factor (TNF)-α has been implicated in the pathogenesis of PD for nearly 30 years since the early finding of a 4-fold increase in TNFα concentrations in the autopsy brains of subjects with PD (Mogi et al., 1994). TNFα knockout mice have an 8-fold reduction in mortality associated with MPTP administration (Ferger et al., 2004). TNFα is secreted in brain by microglial cells, which plays a pro-inflammatory role in the development of human PD (Hamid et al., 2022; Xiromerisiou et al., 2022; Cabrera Ranaldi et al., 2023; Wang et al., 2023). Microglial production of TNFα in PD results in increased secretion of α-synuclein (Bae et al., 2022). If TNFα plays an important role in PD, then the administration of biologic TNF inhibitors (TNFI) should be therapeutic in PD. This was demonstrated in a 6-hydroxydopamine model of experimental PD in the rat. Neuroprotection was produced following the intra-striatal injection of a dominant-negative TNFα analogue, XENP345 (McCoy et al., 2006). XENP345 is a pegylated TNFα variant that binds wild type TNFα to form hetero-trimers that do not bind the TNF receptor (TNFR; Steed et al., 2003). This biologic TNFI had to be administered by intra-cerebral injection, because this large molecule does not cross the BBB. XENP345, also known as XPro®1595, is said to cross the BBB, because the drug was observed to enter the CSF compartment, albeit at a concentration that was 1,000-fold lower than the plasma concentration (Barnum et al., 2014). However, drug distribution into CSF is a measure of transport across the blood-CSF barrier, at the choroid plexus, and not of transport across the BBB, at the brain capillary (Pardridge, 2022b). Biologic TNFIs include etanercept, a Fc-TNFR decoy receptor fusion protein, and TNFα-neutralizing antibodies such as adalimumab or infliximab. Despite the large-scale use of biological TNFIs in inflammatory conditions of peripheral organs (Caporali et al., 2018), these TNFIs have not been successfully developed for PD, or other CNS conditions. The disparity in the use of biologic TNFIs for peripheral conditions versus CNS disease is stunning, considering the global revenue for biologic TNFIs is in excess of $40 billion for peripheral inflammatory conditions, but is zero for the brain. The biologic TNFIs have not been developed as new treatments for PD, or other neurodegenerative conditions, because biologic TNFIs do not cross the BBB. Decoy receptors, such as etanercept, do not cross the BBB (Boado et al., 2010b), and therapeutic antibodies, such as adalimumab or infliximab, do not cross the BBB (Pardridge, 2023a). Successful development of the biologic TNFIs as drugs for the brain requires that these pharmaceuticals are re-engineered to enable transport across the BBB (Pardridge and Boado, 2012). Biologic TNFI decoy receptors can be re-engineered as an IgG-decoy receptor fusion protein that crosses the BBB via RMT as discussed below.

### 3.2. HIRMAb-TNFR fusion protein

**Figure 5:**
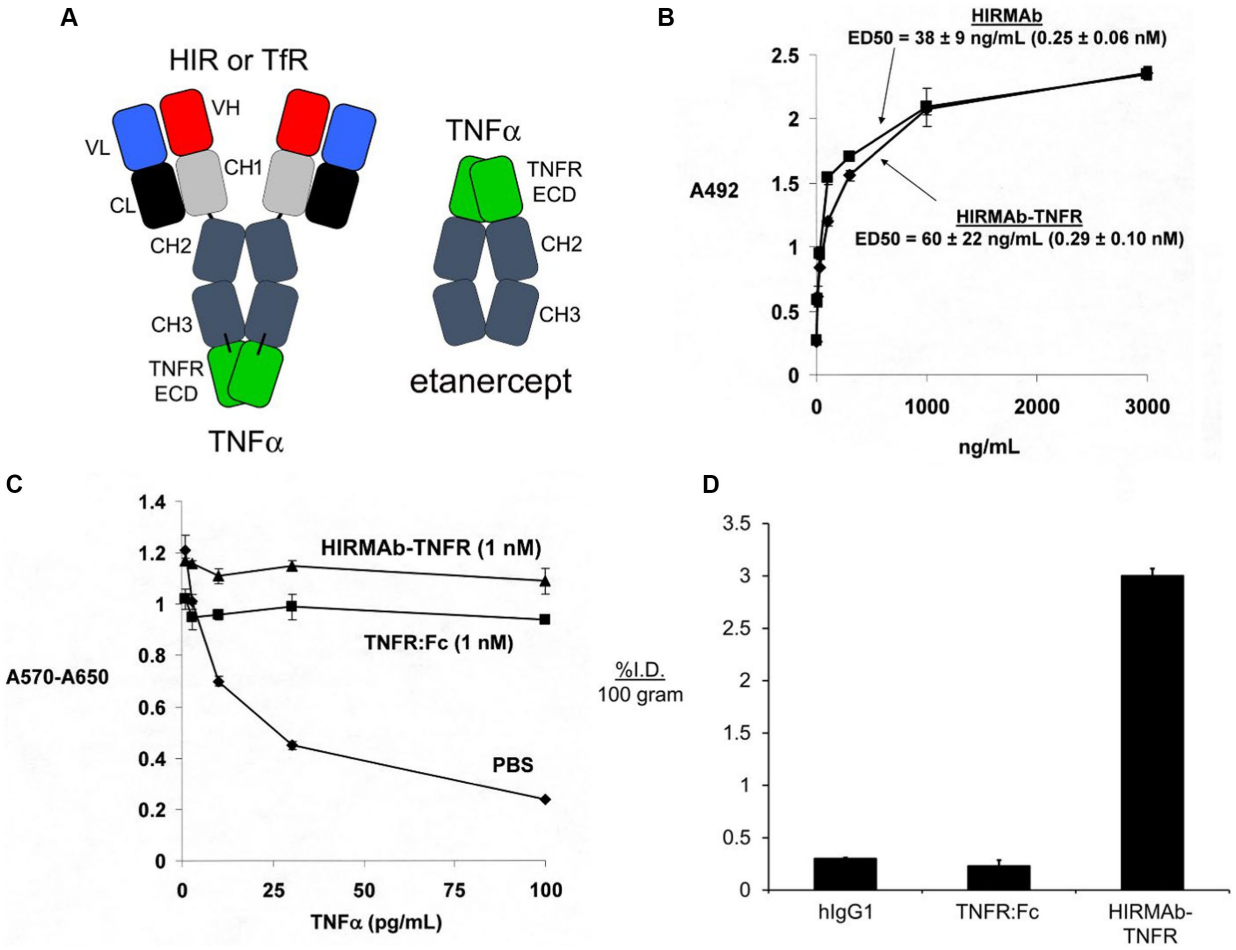
**(A)** Structure of IgG-TNFR2 fusion protein and etanercept. For either molecule, the TNFR2 is the extracellular domain (ECD) of the human TNFRSF1B. The IgG domain is either a HIRMAb, which is active in the primate or human, or a TfRMAb, which is active in the mouse. For etanercept, the TNFR2 ECD is fused to the *amino* terminus of human IgG1 Fc. In contrast, for the HIRMAb-TNFR2, or TfRMAb-TNFR2, fusion protein, the TNFR2 ECD is fused to the *carboxyl* terminus of the CH3 region of the HIRMAb or TfRMAb heavy chain. Reprinted with permission from Zhou et al. (2011d). **(B)** The HIRMAb domain of the HIRMAb-TNFR2 fusion protein retains high affinity binding for the HIR, as there is no difference in ELISA ED50 values for either the HIRMAb-TNFR2 fusion protein or the HIRMAb alone. **(C)** Bio-assay of human TNFα toxicity in human WEHI-13VAR cells treated with 1 ug/mL actinomycin D and exposed to 0–100 pg./mL TNFα in the presence of phosphate buffered saline (PBS), 1 nM etanercept (TNFR:Fc), or 1 nM HIRMAb-TNFR fusion protein. Cytotoxicity was measured with thiazolyl blue tetrazolium bromide. **(D)** Brain uptake, expressed as % injected dose (ID)/100 gram brain, at 2 h after IV administration in the adult Rhesus monkey of [^3^H]-human IgG1, the isotype control antibody for the HIRMAb, [^125^I]-etanercept (TNFR:Fc), or the [^125^I]-HIRMAb-TNFR fusion protein. [^125^I]-radiolabeling was performed with the [^125^I]-Bolton-Hunter reagent. Panels **(B–D)** are reprinted with permission from Boado et al. (2010b).

Etanercept is a fusion protein of human IgG1 Fc and the extracellular domain (ECD) of the TNFR (Peppel et al., 1991), which is superfamily (TNFRSF) member 1B, TNFRSF1B, that binds a trimer of soluble TNFα (Scallon et al., 2002). To re-engineer the TNFRSF1B decoy receptor for BBB RMT delivery, the ECD of the human TNFRSF1B, also called TNFR2, which corresponds to amino acids 23–257 of NP_001057, was fused to the carboxyl termini of both heavy chains of the HIRMAb (Boado et al., 2010b). The structure of the HIRMAb-TNFR fusion protein and etanercept are shown in Figure 5A. In the case of etanercept, the TNFR ECD is fused to the *amino terminus* of each heavy chain of the Fc fragment, whereas in the case of the HIRMAb-TNFR fusion protein, the TNFR ECD was fused to the *carboxyl terminus* of each heavy chain of the HIRMAb. Fusion of the TNFR ECD to the amino terminus of the IgG, as is the case for etanercept, would reduce binding of the fusion protein to the HIR, since the antigen binding variable regions are near the amino terminus of the IgG. Fusion of the amino terminus of the TNFR ECD to the carboxyl terminus of the HIRMAb heavy chain was undertaken because the amino terminus of TNFR2 ECD is not involved in TNFα binding (Banner et al., 1993). The engineering of the HIRMAb-TNFR fusion protein places the TNFR in a dimeric configuration, which replicates the dimeric structure of the native TNFR2 (Chan et al., 2000; Shoji-Hosaka et al., 2006). Following expression of the HIRMAb-TNFR fusion protein in stably transfected CHO cells, the purified HIRMAb-TNFR fusion protein retained high affinity binding to the HIR, as there was no difference in binding of the HIRMAb-TNFR fusion protein, or the HIRMAb alone, to the HIR (Figure 5B). The affinity of the HIRMAb-TNFR fusion protein for TNFα binding was high as the KD of binding in a radio-receptor assay with [^125^I]- TNFα was 0.29 nM (Boado et al., 2010b). A human bio-assay of the cytotoxic effects of TNFα uses the human WEHI-13 VAR cell line exposed to 1 ug/ml actinomycin D (Espevik and Nissen-Meyer, 1986). Both etanercept, also named TNFR:Fc, and the HIRMAb-TNFR fusion protein, at a 1 nM concentration, produced complete cell protection against TNFα in this assay (Figure 5C). The brain uptake, and plasma PK, of etanercept and the HIRMAb-TNFR fusion protein were measured in the adult Rhesus monkey following radio-labeling of each protein. Etanercept was radio-labeled with the [^125^I]-Bolton-Hunter reagent, and the HIRMAb-TNFR fusion protein was radio-labeled with [^3^H]-N- succinimidyl propionate (Boado et al., 2010b). The brain VD of etanercept, 13 ± 3 uL/gram, in the primate is no different from the brain plasma volume, which indicates etanercept does not cross the BBB (Boado et al., 2010b). In contrast, the brain VD of the HIRMAb-TNFR fusion protein was 354 ± 21 uL/gram, and capillary depletion analysis showed the majority of the fusion protein in brain at 2 h after administration was in the post-vascular volume of brain, indicating the fusion protein had fully transcytosed through the BBB (Boado et al., 2010b). The brain uptake of the HIRMAb-TNFR fusion protein, at an intravenous injection dose of 0.2 mg/kg, was 3.0 ± 0.1 %ID/100 grams (Figure 5D). Brain uptake is expressed per 100 grams brain, because the weight of the brain in the Rhesus monkey is 100 grams (Pardridge et al., 1995). The brain uptake of the HIRMAb-TNFR fusion protein in the primate, 3.0 ± 0.1 %ID/100 grams, is high and comparable to the brain uptake of a lipid soluble small molecule. The brain uptake of fallypride, a small molecule dopamine receptor blocker, is about 4% ID/100 grams in the Rhesus monkey following IV administration (Mukherjee et al., 2001). In contrast to the high brain uptake of the HIRMAb-TNFR fusion protein, the brain uptake of etanercept (TNFR:Fc) is very low and is equal to the brain uptake human IgG1, the isotype control of the HIRMAb, which is a marker of the plasma volume in brain (Figure 5D). A biologic confined to the plasma volume of brain does not cross the BBB. The neuroprotective effects of a BBB-penetrating IgG-TNFR fusion protein was tested in an experimental model of moderate PD in the mouse. Since the HIRMAb does not recognize the mouse insulin receptor (Zhou et al., 2012), the TNFR ECD was re-engineered as a TfRMAb-TNFR fusion protein that binds to the murine TfR.

### 3.3. TfRMAb-TNFR fusion protein

**Figure 6:**
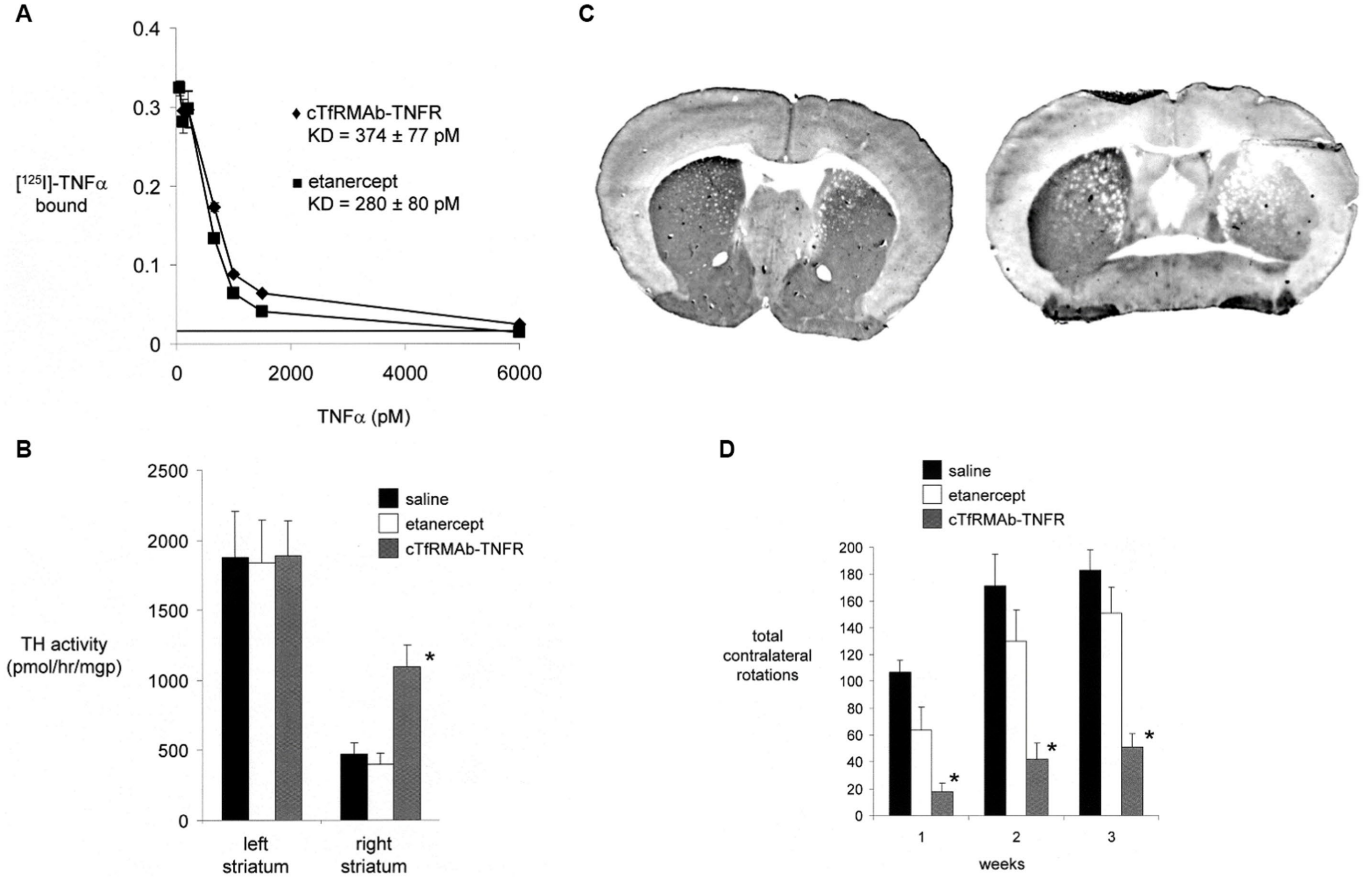
**(A)** Radio-receptor assay shows comparable high affinity binding of [^125^I]-TNFα to either etanercept (closed squares) or the mouse-specific cTfRMAb-TNFR fusion protein (closed diamonds). **(B)** Experimental PD was produced in the mouse following the intra-cerebral injection of 6 ug of 6-hydroxydopamine in each of 2 regions of the right striatum in C57BL/6 J male mice. PD mice were treated every 2 days with saline, 1 mg/kg etanercept IV, or 1 mg/kg cTfRMAb-TNFR fusion protein for 3 weeks, starting 1 h after toxin injection. Toxin administration caused a 75% reduction in tyrosine hydroxylase (TH) enzyme activity in the lesioned striatum, compared to the non-lesioned striatum, and etanercept treatment had no effect on striatal TH enzyme activity. However, treatment with the cTfRMAb-TNFR fusion protein caused a 2.3-fold increase in TH enzyme activity in the lesioned striatum. (*) indicates a statistically significant effect at the *p* < 0.01 level by analysis of variance. **(C)** TH immunohistochemistry (IHC) of coronal sections of PD mouse brain at the end of 3 weeks of treatment is shown for individual mice selected from either the cTfRMAb-TNFR fusion protein treatment group (left panel) or the saline treatment group (right panel). Density scanning of IHC slides for all mice showed the mean reduction in immunoreactive TH in the striatum of the lesioned side was 71% compared to the non-lesioned side, and that treatment with the cTfRMAb-TNFR fusion protein caused a 2.0-fold increase in immunoreactive TH on the lesioned side. **(D)** Apomorphine-induced rotation behavior was measured weekly. Etanercept had no therapeutic effect in the PD model. The normalization of motor activity caused by treatment with the cTfRMAb-TNFR fusion protein was significant at the *p* < 0.01 (*) level at weeks 1, 2, and 3. Data are means±SE (*n* = 10 mice per treatment group). All panels reprinted with permission from Zhou et al. (2011d).

A BBB-penetrating IgG-TNFR fusion protein that is active in the mouse was produced following the fusion of the human TNFR2 ECD to the carboxyl termini of both heavy chains of a mouse/rat chimeric TfRMAb. The chimeric TfRMAb, derived from the rat 8D3 MAb against the mouse TfR, is designated the cTfRMAb, and the fusion protein of this cTfRMAb and the human TNFR2 ECD is designated the cTfRMAb-TNFR fusion protein (Zhou et al., 2011c). The IgG fusion protein formed from the human TNFR2 ECD is expected to be active in the mouse, since etanercept binds human and mouse TNFα with the same high affinity (Scallon et al., 2002). The cTfRMAb-TNFR fusion protein retained high affinity binding to the mouse TfR, and the brain uptake of this fusion protein in the mouse was 2.8 ± 0.5% ID/gram (Zhou et al., 2011c), which is comparable to the brain uptake in the mouse of the cTfRMAb-GDNF fusion protein (Figure 2B). A brain uptake of 3% ID/gram in the mouse is high and comparable to the brain uptake of lipid soluble small molecules. The mouse brain uptake of diazepam, which is freely diffusible through the BBB, is about 5% ID/gram following IV administration (Greenblatt and Sethy, 1990). The uptake of the cTfRMAb-TNFR fusion protein in the cervical, thoracic, and lumbar spinal cord was equal to the brain uptake in the cerebrum (Zhou et al., 2011c). The cTfRMAb-TNFR fusion protein retained high affinity binding for TNFα, as the KD of TNFα binding was the same for either the cTfRMAb-TNFR fusion protein or etanercept (Figure 6A).

The neuroprotective effect of the TfRMAb-TNFR fusion protein in experimental PD was tested in the C57BL/6 male mouse following the intra-cerebral injection of 6 ug of 6-hydroxydopamine in each of 2 regions of the right striatum (Zhou et al., 2011d). Mice were treated by IV administration of saline, 1 mg/kg etanercept, or 1 mg/kg of the TfRMAb-TNFR fusion protein, every 2 days for 3 weeks starting 1 h after toxin injection. This model of experimental PD produced a 75% reduction in striatal TH enzyme activity on the side ipsilateral to the toxin injection. Treatment of the mice with either saline or etanercept produced no change in striatal TH enzyme activity (Figure 6B). However, treatment with the cTfRMAb-TNFR fusion protein produced a 130% increase in striatal TH enzyme activity on the lesioned side (Figure 6B). The results of the TH enzyme activity assay were confirmed by TH IHC of coronal sections of the brain of the PD mice, as shown in Figure 6C. Scanning densitometry of the coronal sections showed the immunoreactive TH in the striatum was reduced 71% by toxin administration in the saline treated mice (Figure 6C, right panel), and that treatment with the cTfRMAb-TNFR fusion protein, on average for all mice, was increased 101% as compared to saline treatment (Figure 6C, right panel). The increase in striatal TH enzyme activity caused by the treatment with the cTfRMAb-TNFR fusion protein was correlated with measurements of motor activity in the mice with PD. The apomorphine-induced rotation behavior was improved 75–83% at 1–3 weeks after toxin administration, whereas etanercept treatment had no significant effect in the mouse model of PD (Figure 6D). The amphetamine-induced rotation behavior was improved 45–67% at 1–3 weeks after toxin administration, whereas etanercept treatment had no significant effect (Zhou et al., 2011d). The vibrissae-elicited forelimb placing test score was reduced 78% in the saline treated PD mice, and this score was increased 82% by fusion protein treatment, whereas etanercept treatment had no therapeutic effect (Zhou et al., 2011d). The ADA titer produced in serum of mice chronically treated with either etanercept or the cTfRMAb-TNFR fusion protein was measured by ELISA. No ADA response was observed in the etanercept treated mice, and only a low titer, <0.1 OD/uL, ADA response was observed in the mice treated with the cTfRMAb-TNFR fusion protein (Zhou et al., 2011d).

In summary, a decoy receptor such as etanercept, or other decoy receptors that also block pro-inflammatory cytokines (Kefaloyianni, 2022), can be re-engineered for brain drug delivery via RMT across the BBB. Instead of fusion of the TNFRSF1B ECD to human Fc, the receptor ECD is fused to the carboxyl terminus of the heavy chain, or the light chain, of a MAb that undergoes RMT across the BBB, as outlined in Figure 5A. Neuroinflammation plays an important role in the pathogenesis of PD (Wang et al., 2015; Tansey et al., 2022), and cytokines other than TNFα play a part in this inflammation, including interleukin (IL)-1β (Wang et al., 2015) or IL-4 (Bok et al., 2018). IL1β activity in brain could be suppressed by re-engineering the IL1-Trap fusion protein, rilonacept (Hoffman et al., 2008), for BBB transport. IL4 activity in brain could be suppressed by re-engineering the IL4-Trap fusion protein, altrakincept (Hendeles et al., 2004), for BBB transport. Alternatively, cytokine action in brain may be suppressed by the re-engineering of cytokine-binding monoclonal antibodies, such as adalimumab or infliximab, which bind TNFα, or cankinumab, which binds IL1β, as bispecific antibodies that penetrate the BBB via RMT, as discussed in the next section.

## 4. Blood–brain barrier receptor-mediated transport of therapeutic bi-specific antibodies in Parkinson’s disease

The pathologic hallmark of PD is the Lewy body, which is formed by α-synuclein (SYN) aggregates (Rodger et al., 2023). In parallel with the development of anti-Abeta amyloid antibodies (AAA) for Alzheimer’s disease (AD), anti-SYN therapeutic antibodies have entered clinical trials for the treatment of PD. Since therapeutic antibodies do not cross the BBB (Pardridge, 2023a), the failure of the anti-SYN clinical trials in PD might be anticipated. Therapeutic antibodies for the CNS that have received FDA approval include antibodies for multiple sclerosis (MS), glioma, and AD. However, the therapeutic antibodies that are approved for MS or glioma have a site of action within the blood compartment, not the brain, and BBB transport is not required. Therapeutic antibodies for MS that target proteins behind the BBB, which does necessitate antibody transport across the BBB, have failed in clinical trials (Pardridge, 2023a). In contrast, the AAAs for AD target brain amyloid, which is behind the BBB, and these AAAs must traverse the BBB to reduce brain amyloid. These AAAs have a special property of causing BBB disruption in subjects with AD, and this BBB disruption allows the AAA to gain access to amyloid plaques behind the BBB. A unique property of the therapeutic AAAs in AD is the development of amyloid related imaging abnormality (ARIA) following AAA treatment in AD. The reduction in amyloid plaque in AD caused by AAA treatment is directly proportional to the ARIA induced by AAA treatment in clinical trials (Wang et al., 2022). ARIA is a measure of brain edema and BBB disruption induced by AAA treatment. Since there is no evidence that anti-SYN antibodies cause BBB disruption, there is no mechanism by which these therapeutic antibodies may gain access to brain following systemic administration. Multiple anti-SYN antibodies have entered clinical trials in PD. Last year, the clinical trial failures of cinpanemab and prasineuzumab anti-SYN antibodies for PD were reported (Lang et al., 2022; Pagano et al., 2022). Other anti-SYN antibodies to enter clinical trials for PD include ABBV-0805 (Nordstrom et al., 2021), MEDI1341 (Schofield et al., 2019) and UCB7853. The ABBV-0805 anti-SYN antibody trial in PD was stopped by the sponsor (Alzforum, 2023a). Similarly, the UCB7853 anti-SYN antibody trial in PD was discontinued by the sponsor (Alzforum, 2023b). The editorial (Whone, 2022) summarizing the reports of the cinpanemab and prasineuzumab trial failures made no mention of the issue of the BBB, or whether the clinical trial failure was due to inadequate delivery of the drug, rather than the intrinsic therapeutic action of the anti-SYN antibody in PD. It is anticipated that drug development of anti-SYN therapeutic antibodies for PD will follow the same course as GDNF drug development for PD. GDNF is no longer in active drug development for PD, because the prior clinical trials of GDNF for PD failed (Nutt et al., 2003; Lang et al., 2006). Already, the development of an α-synuclein blocking antibody as a therapeutic for PD has been recently questioned, based on the failed clinical trials (Patani et al., 2023). However, failed clinical trials provide no insight into the efficacy of a drug, if that pharmaceutical for PD does not reach the target site in brain following drug administration. A failed clinical trial should prompt PD drug developers to question the viability of the brain delivery system, before doubts are raised about the therapeutic efficacy of the drug. The section below proposes that anti-SYN antibodies, or any therapeutic antibody, might succeed as a new treatment for PD, providing the therapeutic antibody is re-engineered for RMT delivery across the BBB. Since the delivery agent is a receptor-specific antibody, and the therapeutic agent is also an antibody, the goal is the engineering of a new bi-specific antibody (BSA) that both targets a receptor on the BBB, to enable delivery across the BBB, and targets the neural protein behind the BBB, to enable therapeutic action in brain.

### 4.1. Blood–brain barrier receptor-mediated transport of α-synuclein bi-specific antibodies in Parkinson’s disease

An anti-SYN therapeutic antibody for PD has been re-engineered as a BBB-penetrating BSA that undergoes RMT across the BBB via either the TfR (Roshanbin et al., 2022) or the IGFR (Shin et al., 2022). In the case of the TfR-directed BSA (Roshanbin et al., 2022), the domain targeting SYN was derived from the Syn-02 antibody, which binds SYN aggregates, but not soluble SYN (Vaikath et al., 2015). A single chain Fv (scFv) antibody was derived from the 8D3 rat MAb against the mouse TfR, and this scFv was fused to the carboxyl terminus of each light chain of the Syn-02 antibody, and this BSA was designated AbSyn02-scFv8D3 (Roshanbin et al., 2022). The therapeutic effects of the AbSyn02-scFv8D3 BSA was assessed in the L61 mouse, which over-produces SYN aggregates (Rockenstein et al., 2002). The L61 mouse was treated with 10 mg/kg of either the Syn-02 antibody alone, or the AbSyn02-scFv8D3 BSA, on days 1, 2, and 4, followed by euthanasia on day 5. This short course produced a modest reduction of SYN aggregates in brain solubilized with 0.5% Triton X-100 in the L61 mice treated with the BSA, but there was no effect of the Syn-02 antibody on SYN aggregates in brain (Roshanbin et al., 2022). It is expected that a longer duration of treatment with a SYN targeting BBB-crossing BSA will produce larger reductions in SYN aggregates in brain in models of PD.

A MAb against human SYN, designated M30103, was re-engineered for BBB penetration by fusion of a scFv antibody against the human IGF1 receptor (IGF1R) to the carboxyl terminus of one heavy chain of the M30103 antibody to produce the BSA, designated B30104 (Shin et al., 2022). The anti-IGF1R scFv, designated Grabody B, was reactive across species, which allowed for testing in mouse models of PD. The choice to engineer the BBB-directed arm of the BSA in a monovalent format insured low affinity of the BSA for the BBB IGF1R. As the affinity of the BSA for the BBB receptor is reduced, the therapeutic injection dose (ID) of the BSA must be proportionately increased (Pardridge, 2023a). The 6-hydroxydopamine or MPTP rodent models of experimental PD do not generate SYN aggregates that comprise the Lewy bodies of human PD (Tieu, 2011; Paul and Sullivan, 2019). Therefore, a SYN aggregate model of PD in the mouse was produced following the intra-cerebral injection of pre-formed fibrils (PFF) of human SYN (Volpicelli-Daley et al., 2011). At 1 week following the intra-cerebral injection of the PFFs, the mice were treated for 6 months by weekly intra-peritoneal injections of 15–18 mg/kg of either the M30103 anti-SYN antibody or the B30104 BBB-penetrating BSA (Shin et al., 2022). This dose is 10-fold higher than the dose, 1 mg/kg, used in mouse models of neural disease with BBB-penetrating biologics derived from a MAb that binds the RMT system on the BBB via high affinity bivalent binding (Pardridge, 2023a). As a consequence of the high injection dose, treatment with either the M303013 antibody alone, or with the B30104 BSA, caused a reduction in SYN aggregates in brain (Shin et al., 2022). Engineering of a tetravalent BSA with high affinity for both SYN aggregates, and the BBB RMT system, may enable the selective reduction of SYN aggregates in experimental PD at therapeutic injection doses, 1 mg/kg, of the BSA.

### 4.2. Blood–brain barrier receptor-mediated transport of neurotrophin receptor agonist bi-specific antibodies in Parkinson’s disease

Neurotrophins such as GDNF or brain derived neurotrophic factor (BDNF) are potential biologics that can facilitate repair of dystrophic neurons in brain in PD, should the neurotrophin be enabled to cross the BBB. In a clinical trial of amyotrophic lateral sclerosis, recombinant human BDNF was administered by subcutaneous (SC) administration (BDNF Study Group, 1999). The clinical trial failed, because BDNF does not cross the BBB. An alternative to BDNF as a biologic for PD is an agonist antibody for the BDNF receptor, which is the tyrosine kinase receptor (TrkB). The 29D7 MAb binds the human or mouse TrkB with agonist activity in the low nM range (Qian et al., 2006). This antibody was re-engineered as a BBB-penetrating BSA. A single chain shark variable domain of new antigen receptor (VNAR) antibody, designated TXB4, binds the mouse or human TfR with high affinity, and undergoes RMT across the BBB (Clarke et al., 2022). A BSA was genetically engineered, where the transporting antibody was derived from the TXB4 VNAR anti-TfR antibody, and the therapeutic antibody was derived from the 29D7 TrkB agonist antibody. A mouse model of experimental PD with modest degeneration was produced with a single intra-striatal injection of 4 ug of 6-hydroxydopamine (Clarke et al., 2022). PD mice were treated with SC injections of 2.5–5 mg/kg of the TXB4-TrkB BSA, or the 29D7 MAb alone, at −1 and + 7 days relative to toxin injection in brain, and were euthanized at 14 days after toxin injection for measurement of immunoreactive TH in the substantia nigra. The number of cell bodies immunoreactive for TH was reduced 27 ± 7% and 3 ± 2% in the saline and BSA treated animals, respectively, in the substantia nigra on the lesioned side, relative to the nigral cell count on the non-lesioned side (Clarke et al., 2022). Treatment with the 29D7 antibody alone had no significant neuroprotective effect. This preliminary study suggests that agonist antibodies for neurotrophin receptors, such as TrkB, or even GFRα1, are potential new treatments for PD, should these agonist antibodies be re-engineered for RMT delivery across the BBB.

## 5. Glucagon-like peptide-1 and Parkinson’s disease

Glucagon-like peptide 1 (GLP1) is a hormone secreted by the gut after a meal, and GLP1 agonists are now widely used as a treatment for type 2 diabetes mellitus, following the 2005 FDA approval of the first in class, exenatide, a 39 amino acid synthetic form of exendin-4, a GLP1-mimetic peptide that binds the GLP1 receptor (Maselli and Camilleri, 2021). The ICV infusion of exendin-4 as a pre-treatment of MPTP-induced PD in the mouse was neuroprotective (Li et al., 2009). Subsequently, clinical trials of weekly SC injections of exenatide in PD were performed, although a review of these trials found the evidence for a beneficial effect in PD to be of low certainty (Mulvaney et al., 2020). The Exenatide-PD3 trial was initiated in 2020 (Vijiaratnam et al., 2021), with no results posted to date (NCT04232969). If GLP1 agonist peptides are therapeutic in PD following SC administration, then it would be necessary for the peptide to undergo RMT across the BBB via the GLP1 receptor (GLP1R) expressed at the BBB. The GLP1R was cloned over 30 years ago (Thorens, 1992), and following immunization of mice with the human GLP1R ECD, a highly specific anti-GLP1R MAb, 3F52, was developed (Pyke et al., 2014). Immunohistochemistry of brain with the MAb3F52 shows immunoreactive GLP1R on brain cells, but not on brain endothelium (Heppner et al., 2015). Expression of the GLP1R at the brain capillary would be necessary for RMT delivery of GLP1 from blood to brain. The extent to which exendin-4 crosses the BBB following IV administration was evaluated in mice (Salameh et al., 2020). The peptide was radio-iodinated with chloramine T, in parallel with the labeling of bovine serum albumin with ^99m^Tc (technetium), a marker of the brain plasma volume (Vo). The brain volume of distribution of exendin-4 was barely above the Vo of BSA, and the minor brain uptake of exendin-4 was not saturable (Salameh et al., 2020), which indicates the negligible BBB transport of exendin-4 is not receptor-mediated. The absence of an immunoreactive GLP1R at the BBB, and the absence of saturable transport of exendin-4 across the BBB *in vivo*, indicate there is no RMT system at the BBB to enable delivery of GLP1 agonists from blood to brain. If this is the case, then treatment of PD patients with exenatide may prove to be of no benefit.

## 6. Non-viral gene therapy of Parkinson’s disease with plasmid DNA encapsulated in Trojan horse lipid nanoparticles

The development of gene therapeutics for PD, as is the case for gene therapy of any disease (Kuzmin et al., 2021), is >99% based on the use of viral vectors, mainly adeno-associated virus (AAV). For PD, the AAV vector is delivered to brain via convection enhanced diffusion (Van Laar et al., 2021). The limitations of AAV gene therapy of brain have been reviewed previously (Pardridge, 2022b). Given the challenges in AAV gene therapy of brain disorders, it is important to develop, in parallel, non-viral technologies for the delivery to brain of plasmid DNA encoding therapeutic genes. This is possible following the encapsulation of the large nucleic acid within the interior of a pegylated liposome type of lipid nanoparticle (LNP). The encapsulation of a 4 kb mRNA within pegylated liposomes was used to produce, at scale, the COVID19 LNP vaccines (Corbett et al., 2020; Sahin et al., 2020). In addition to large size mRNA, it is also possible to encapsulated plasmid DNA within pegylated liposomes (Monnard et al., 1997; Wheeler et al., 1999). However, pegylated liposomes do not cross the BBB (Pardridge, 2023b). Pegylated liposomes, encapsulated with plasmid DNA, can be enabled to penetrate the BBB by conjugation of receptor-specific antibodies to the surface of the LNP. The MAb on the surface of the LNP targets an endogenous BBB receptor, such as the insulin receptor or the transferrin receptor, and acts as a molecular Trojan horse to trigger RMT of the antibody-modified LNP across the BBB (Pardridge, 2023b). The LNP conjugated with the receptor-specific MAb is designated a Trojan horse liposome (THL), and the IV administration of THLs encapsulating plasmid DNA encoding the lacZ reporter gene is followed by global expression of the lacZ gene within the brain of mice, rats, and monkeys (Pardridge, 2023b). THLs have also been produced with therapeutic genes for PD including plasmid DNA encoding either TH or GDNF, as reviewed below.

### 6.1. Blood–brain barrier receptor-mediated transport in Parkinson’s disease of plasmid DNA encoding tyrosine hydroxylase under the influence of a brain-specific promoter encapsulated in Trojan horse LNPs

**Figure 7:**
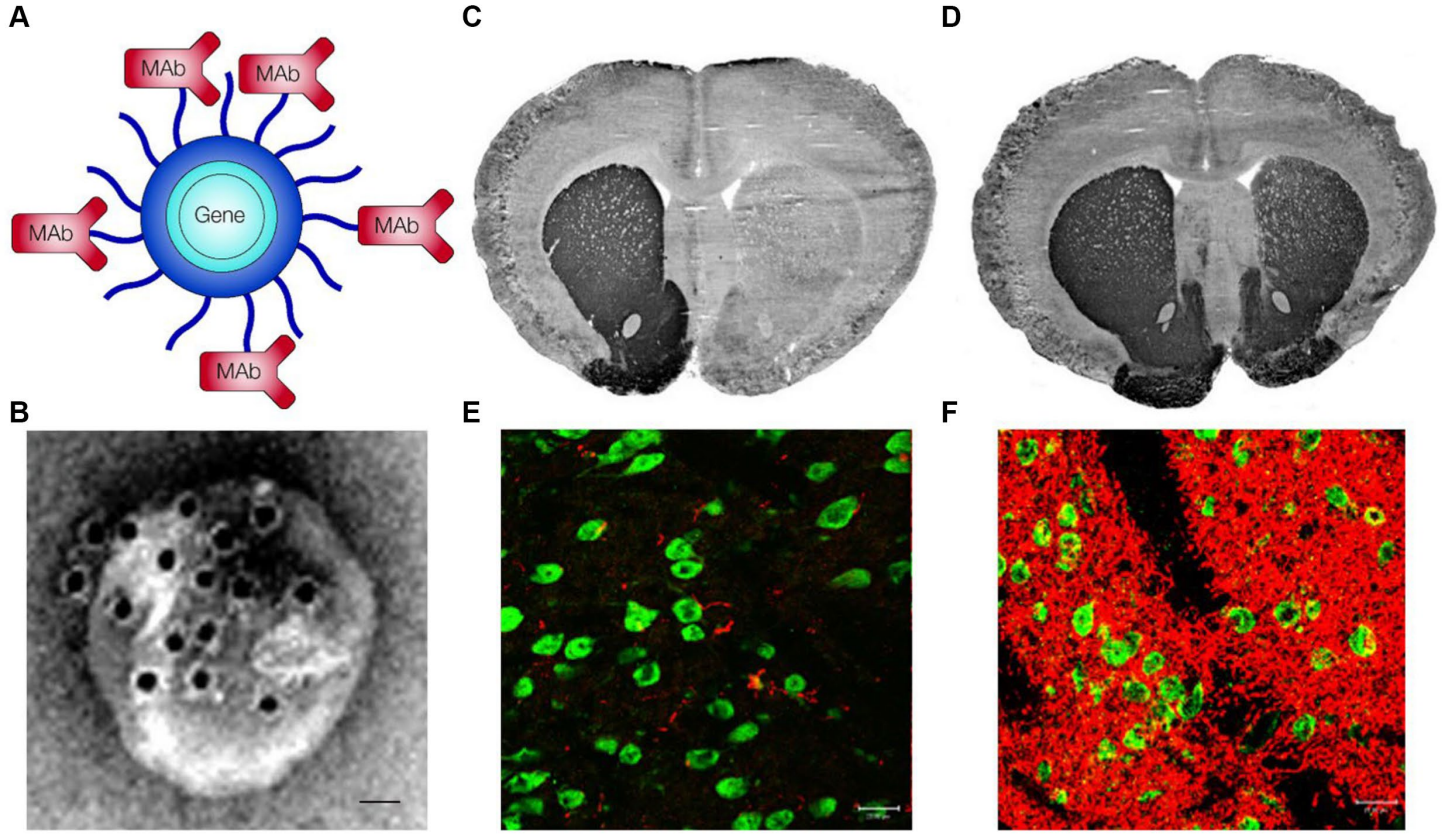
**(A)** Structure of a Trojan horse lipid nanoparticle (LNP), also called a Trojan horse liposome (THL), shows the expression plasmid DNA encapsulated in the interior of the LNP and the receptor-specific MAb conjugated at the tips of 2,000 Da polyethylene glycol strands on the surface of the LNP. For treatment of rats with experimental PD, the MAb was either the mouse OX26 MAb against the rat TfR, or the mouse IgG2a isotype control antibody. Reprinted with permission from Pardridge (2002). **(B)** Transmission electron micrograph of a THL complexed with a conjugate of a secondary antibody and 10 nm gold particles. Magnification bar = 20 nm. Reprinted with permission from Zhang Y. et al. (2003). **(C,D)** Tyrosine hydroxylase (TH) immunohistochemistry of coronal sections of brain removed from adult Sprague–Dawley rats injected with 8 ug of 6-hydroxydopamine in the right median forebrain bundle. Brains were removed 72 h after the IV administration of 10 ug plasmid DNA/rat encapsulated in LNPs conjugated with either mouse IgG2a control antibody **(C)** or the OX26 rat TfRMAb **(D)**. The plasmid DNA expressed rat TH under the influence of a brain-specific glial fibrillary acidic protein (GFAP) promoter. Treatment with the THLs was initiated at 3 weeks after toxin administration. **(E,F)** Confocal microscopy of the striatum on the lesioned side of the brains described in panels **(C,D)**, respectively, where the brain was stained with antibodies against the neuronal nuclear protein, neuN (green channel) or TH (red channel). Panels **(E,F)** show the lesioned striatum in the rats treated with mouse IgG2a-THLs or OX26-THLs, respectively. Panels **(C–F)** reprinted with permission from Zhang et al. (2004).

The structure of a THL is outlined in Figure 7A, which shows encapsulation of the plasmid DNA in the interior of a 100 nm pegylated liposome, where 1–2% of the 2,000 Da polyethylene glycol (PEG) strands on the surface of the liposome is conjugated with a receptor-specific MAb. The MAb targets an endogenous BBB RMT system, such as the insulin receptor or TfR. The THL is visualized with transmission electron microscopy (EM) following complexation of the THL with a gold-conjugated secondary antibody (Figure 7B). The size of the 10 nm gold particles approximates the size of a MAb molecule, and the EM shows the MAb extended from the surface of the THL via the PEG strands. THL-mediated gene therapy of experimental PD in the rat was tested with THLs conjugated with the OX26 mouse MAb against the rat TfR, which encapsulated a plasmid DNA that encoded rat tyrosine hydroxylase (TH). Experimental PD was produced in the rat following the intra-cerebral injection of 8 ug of 6-hydroxydopamine into the right median forebrain bundle, MFB (Zhang et al., 2004). A plasmid DNA encoding the rat TH cDNA under the influence of the widely expressed SV40 promoter was engineered (Zhang Y. et al., 2003). In addition, a TH expression plasmid was engineered where the TH cDNA was under the influence of a brain-specific promoter taken from the 2.2 kb of the 5′-flanking sequence (FS) of the human glial fibrillary acidic protein (GFAP) gene (Genbank M67446). The 3′-untranslated region (UTR) of the TH expression plasmid included 200 nucleotides from the bovine GLUT1 glucose transporter mRNA as a stabilizing element (Zhang et al., 2004), which increased by several-fold the expression of the TH mRNA in transfected cells (Zhang Y. et al., 2003). The 5’-FS of the GFAP gene confers brain specificity of transgene expression, but not astrocyte-specific expression. Astrocyte specific gene expression requires the coordinated interaction of both the 5’-FS and the 3’-FS of the GFAP gene (Kaneko and Sueoka, 1993; Zhuo et al., 2001). The GFAP-TH expression plasmid, designated clone 951, was encapsulated within 100 nm pegylated liposomes, and the surface of these LNPs was conjugated with either the mouse OX26 MAb against the rat TfR, or with the mouse IgG2a isotype control antibody. These Trojan horse LNPs, encapsulated with the GFAP-TH expression plasmid DNA, were designated as OX26-THLs and mIgG2a-THLs, respectively. The OX26-THL and mIgG2a-THL formulations were identical with the exception that the mIgG2a-THLs had no affinity for the rat TfR. The THLs were injected IV in PD rats at a single dose of 10 ug plasmid DNA/rat at 4 weeks following intra-cerebral injection of the 6-hydroxydopamine. The rats were euthanized 3 days following the single IV injection of the THLs for measurement of TH enzyme activity in the striatum, immunoreactive TH by IHC or confocal microscopy, and apomorphine-induced rotation behavior. The intra-cerebral injection of 6-hydroxydopamine into the MFB produces a more severe model of PD as compared to the intra-striatal injection of the toxin (Tieu, 2011; Jagmag et al., 2015). The striatal TH enzyme activity was reduced 98% at 4 weeks following the toxin injection into the MFB in the rat (Zhang et al., 2004), as compared to the 78% reduction in striatal TH enzyme activity following the intra-striatal injection of the toxin in the mouse (Figure 4B). The administration of mIgG2a-THLs had no effect on striatal TH enzyme activity on the lesioned side; in contrast, administration of OX26-THLs produced a 100% restoration of striatal TH enzyme activity (Zhang et al., 2004). The 98% reduction in striatal TH enzyme activity on the lesioned side was correlated with TH IHC on coronal sections of brain removed 3 days after the IV administration of either mIgG2a-THLs or OX26-THLs encapsulating the GFAP-TH expression plasmid. The nearly complete loss of immunoreactive striatal TH in the MFB-injected model of PD is shown by the IHC in Figure 7C, which compares the immunoreactive TH in the lesioned right striatum with the non-lesioned left striatum. The IHC shown in Figure 7C was performed on a rat treated with the mIgG2a-THLs, and shows that these THLs, which are not targeted to the BBB TfR, have no therapeutic effect in the model of experimental PD. In contrast, the IHC shown in Figure 7D was performed on a rat with PD treated with OX26-THLs, and this study shows a complete restoration of immunoreactive TH in the striatum of the lesioned rats. The results with IHC were confirmed with confocal microscopy of the striatum in the rats treated with mIgG2a-THLs (Figure 7E) or with OX26-THLs (Figure 7F). There is abundant immunoreactive TH in the nerve fibers within the striatum of the rats treated with OX26-THLs. The restoration of striatal TH in the PD rats was also reflected in the apomorphine-induced rotation behavior of the rats with experimental PD. The apomorphine-induced rotation was 22 ± 3 rotations/min (RPM) and 4 ± 3 RPM in the rats treated with mIgG2a-THLs and the OX26-THLs, respectively, at 3 days following THL administration (Zhang et al., 2004). It was possible to normalize striatal TH levels at 4 weeks following toxin injection with OX26-THL administration, because there is intense sprouting of surviving dopaminergic neurons from the substantia nigra to the striatum, which begins soon after intra-cerebral 6-hydroxydopamine injection (Finkelstein et al., 2000; Parish et al., 2002). The confocal microscopy shown in Figures 7E,F shows co-labeling of brain with antibodies to TH and the neuN neuronal marker. In addition, co-labeling was performed with antibodies to TH and GFAP (Zhang et al., 2004). No expression of TH in astrocytes was observed, and this was attributed to the absence of expression of GTP cyclohydrolase, GTPCH, in astrocytes (Nagatsu et al., 1995; Hwang et al., 1998). GTPCH is the rate-limiting enzyme in the production of tetrahydrobiopterin (BH4), which is a co-factor for the TH enzyme. Cellular expression of TH is dependent on the parallel cell expression and production of the GTPCH enzyme and BH4 co-factor (Bowling et al., 2008; Ichinose et al., 2008; Homma et al., 2013). Ectopic expression of the TH transgene was not observed in the cortex of the OX26-THL treated rats (Zhang et al., 2004). The small amount of TH activity in the cortex of rats arises from cortical inter-neurons (Benavides-Piccione and DeFelipe, 2007), which migrate to the cortex (Wonders and Anderson, 2006). Ectopic expression of the TH transgene was observed in liver if the TH gene was under the influence of the SV40 promoter, but this ectopic expression in liver was eliminated with the use of the GFAP promoter (Zhang et al., 2004).

### 6.2. Blood–brain barrier receptor-mediated transport in Parkinson’s disease of plasmid DNA encoding GDNF under the influence of a tyrosine hydroxylase promoter encapsulated in Trojan horse LNPs

**Figure 8:**
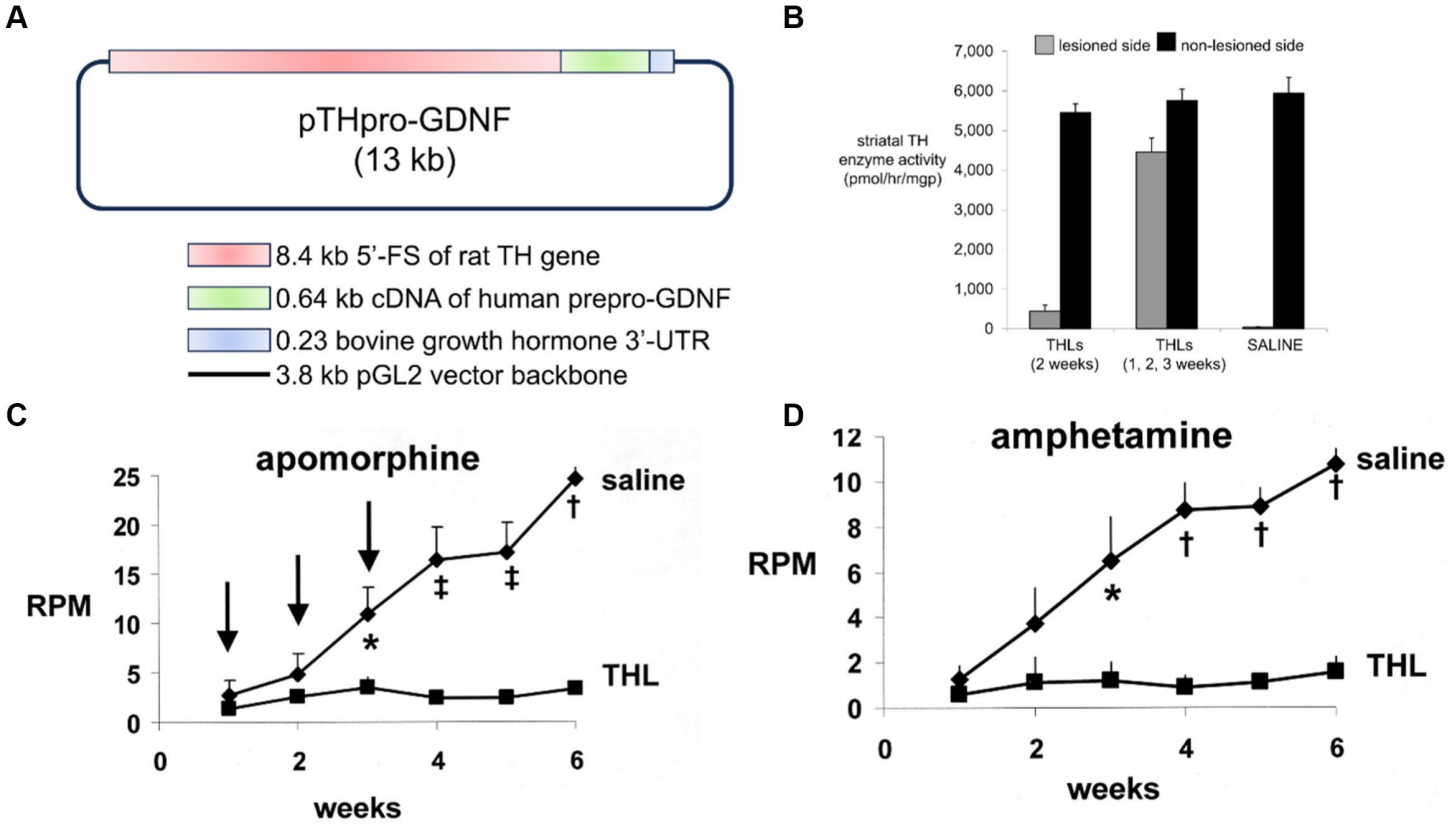
**(A)** The expression cassette of the 13 kb pTHpro-GDNF plasmid includes the 8.4 kb rat TH genomic 5’flanking sequence (FS), the 0.64 kb human preproGDNF cDNA, and the 0.23 kb bovine growth hormone 3′-untranslated region (UTR), as described by Xia et al. (2008). **(B)** Tyrosine hydroxylase (TH) enzyme activity in the striatum of the lesioned and non-lesioned sides of brain from rats administered 8 ug of 6-hydroxydopamine in the right median forebrain bundle. PD rats were treated with saline, with a single dose of OX26-THLs at 2 weeks after the lesion, or with a total of 3 doses of OX26-THLs each at 1, 2, and 3 weeks after the lesion. All rats were euthanized at 6 weeks after toxin administration for measurement of striatal TH enzyme activity. The OX26-THLs encapsulated the pTHproGDNF plasmid DNA and the dose of encapsulated DNA was 10 ug/rat. **(C,D)** Apomorphine-induced **(C)** and amphetamine-induced **(D)** rotation behavior measured weekly after toxin administration at zero time. Rotation is measured as rotations/min (RPM) and include only complete 360° rotations. The arrows show the treatment of the PD rats at weeks 1, 2, and 3 with either saline or OX26-THLs encapsulated with pTHproGDNF. Statistical differences are measured at the *p* < 0.05 (*), *p* < 0.005 (‡), and *p* < 0.0005 (†) at 3–6 weeks after toxin administration. Panels **(B–D)** from Zhang and Pardridge (2009).

The delivery of plasmid DNA to brain with Trojan horse LNPs does not lead to long-lasting gene expression in brain, owing to the degradation in brain of the episomal plasmid DNA (Chu et al., 2006). The brain TH enzyme activity decays with a half-time of 3 days following the administration of OX26-THLs encapsulated with a plasmid encoding TH (Zhang Y. et al., 2003). TH replacement therapy is not needed in PD if neurotrophin gene therapy produces a long-lasting restoration of nigral-striatal neurons in PD. GDNF is a potent neurotrophic factor for the nigral-striatal cells (Lin et al., 1993). Therefore, an expression plasmid was engineered that encoded the 211 amino acid human preproGDNF (Genbank NM_000514). So as to restrict transgene expression to the nigral-striatal tract, the GDNF cDNA was placed under the influence of the 8.4 kb of the 5’-FS of the rat TH gene, as outlined in Figure 8A. This 13 kb expression plasmid, designated pTHproGDNF, includes the bovine growth hormone (BGH) poly A sequence at the 3’-UTR (Xia et al., 2008). Plasmid DNA as large as 22 kb can be successfully encapsulated in THLs (Xia et al., 2007). The full 8 kb of the 5’-FS of the rat TH gene was used as prior work showed this 7–9 kb of the rat TH 5’-FS was necessary to confer tissue specific gene expression within catecholaminergic neurons including the substantia nigra (Wang et al., 1999; Chun et al., 2002). The therapeutic activity of the pTHproGDNF in experimental PD was assessed following the intra-cerebral injection of 8 ug of 6-hydroxydopamine in the right MFB of adult rats. Animals were treated with either saline or 10 ug plasmid DNA of pTHproGDNF encapsulated in OX26-THLs at either 2 weeks after toxin injection as a single administration, or as 3 weekly IV injections at 1, 2, and 3 weeks after toxin administration (Zhang and Pardridge, 2009). Apomorphine-induced and amphetamine-induced rotation behavior was determined weekly for 6 weeks after toxin injection, and rats were euthanized at 6 weeks for measurement of striatal TH enzyme activity. In this model of PD, striatal TH enzyme activity was reduced 99% in the saline treated animals (Figure 8B). Striatal TH enzyme activity was reduced 91% in the animals treated with a single dose of THLs at 2 weeks. Striatal TH enzyme activity was reduced only 23% at 6 weeks in the rats treated with THLs at 1, 2, and 3 weeks after toxin injection (Figure 8B). There was a progressive increase in apomorphine- and amphetamine-induced rotational activity over 6 weeks after toxin injection in the saline treated rats (Figures 8C,D). However, in the rats treated with OX26-THLs encapsulated with pTHproGDNF at 1, 2, and 3 weeks, there was a sustained reduction in drug-induced rotation behavior (Figures 8C,D). The apomorphine-induced rotational activity at 6 weeks was reduced 87% from 25 ± 2 RPM in the saline treated rats to 3 ± 1 RPM in the OX26-THL treated animals. The amphetamine-induced rotational activity at 6 weeks was reduced 90% from 11 ± 1 RPM in the saline treated rats to 1.1 ± 0.2 RPM in the OX26-THL treated animals. GDNF gene therapy, as compared to TH replacement gene therapy, produces a longer lasting therapeutic effect in experimental PD. Ectopic gene expression can be minimized with the use of a tissue-specific gene promoter, such as the 8 kb of the TH 5’-FS, that largely restricts GDNF gene expression to the substantia nigra in brain.

One advantage of non-viral gene therapy with THLs, as compared to AAV viral vectors, is that plasmid DNA as large as 22 kb can be encapsulated in THLs (Xia et al., 2007), whereas expression cassettes for AAV vectors is restricted to <2.3 kb and < 4.7 kb for self-complementary and single stranded vectors, respectively (Pardridge, 2023b). The translation of non-viral gene therapy with THLs requires future work demonstrating the safety of chronic treatment with THLs and the scalability of THL manufacturing. Regarding potential toxicity, rats were treated weekly for 6 consecutive weeks with saline, mIgG2a-THLs, or OX26-THLs encapsulated with a 6 kb rat TH expression plasmid DNA at a dose of 5 ug/rat of THL encapsulated plasmid DNA (Zhang Y. F. et al., 2003). Treatment produced no differences in body weights, 14 serum chemistries, or organ histology of brain, liver, spleen, kidney, heart or lung. IHC with multiple antibodies related to immune function showed no inflammation in brain. Brain uptake of the plasmid DNA was confirmed by Southern blotting (Zhang Y. F. et al., 2003). With regard to scalability of THL manufacturing, the Covid-19 mRNA/LNP vaccines were produced at scale with an ethanol dilution method (Corbett et al., 2020; Sahin et al., 2020). This same ethanol dilution method can be applied to scalable manufacture of THLs (Pardridge, 2023b).

## 7. Perspective

The pathology of PD is characterized by the neurodegeneration of dopaminergic neurons in the nigral-striatal tract in parallel with the accumulation of intra-neuronal α-synuclein aggregates, a process that is exacerbated by neuro-inflammation and the release of pro-inflammatory cytokines in brain by microglia and reactive astrocytes (Wang et al., 2015; Lyra et al., 2023; Schonhoff et al., 2023). Therefore, the future treatment of the neurodegeneration of PD may require combination therapy that (a) repairs dystrophic neurons, e.g., with neurotrophin therapy, (b) reduces neuro-inflammation, e.g., with cytokine blocking decoy receptors, and (c) disaggregates α-synuclein, e.g., with monoclonal antibodies targeting domains of α-synuclein. The neurodegenerative changes leading to symptomatic PD begins in the prodromal phase of PD, which may begin years before the appearance of motor changes (Siderowf and Lang, 2012; Hustad and Aasly, 2020; Berg et al., 2021). The neurodegeneration of PD progresses through 6 stages (Braak et al., 2003). The prodromal period corresponds to stages 1–2, and motor symptoms, which lead to the diagnosis of PD, generally appear in stage 3 (Koeglsperger et al., 2023). The initiation of BBB-penetrating biologics at stage 3 may arrest the disease and slow, or prevent, progression through stages 4–6. The development of PD-specific biomarkers will provide the opportunity for diagnosis during the prodromal period (Filippi et al., 2023), as well as for early initiation of biologics therapy.

Biologics have the potential to treat all 3 components of PD pathology, and repair dystrophic neurites, reduce neuro-inflammation, and disaggregate α-synuclein deposits. Neurotrophic factors, such as GDNF or EPO, can facilitate the repair of dystrophic neurons. Decoy receptors, such as the TNFR ECD, can block that action of pro-inflammatory cytokines in brain such as TNFα. Therapeutic antibodies targeting α-synuclein can lead to disaggregation of α-synuclein intra-neuronal inclusions. The central problem, however, with the treatment of PD, or any brain disease, with biologics is that these large molecule pharmaceuticals do not cross the BBB. Given the limiting role of the BBB in the development of new therapeutics for PD, one could imagine that the development of BBB drug delivery technology would be a central component of the PD drug development process. However, this is not the case. The PD drug development effort within either academic or industry centers is characterized by a minimal effort in BBB drug delivery. The belief that new drugs can be developed for PD, in the absence of any consideration to BBB drug delivery, is illustrated by an analysis of the literature. PubMed lists 73,898 citations under the search term, ‘Parkinson’s disease treatment,’ but only 401 citations, or 0.5%, under the search term, ‘Parkinson’s disease treatment and blood–brain barrier drug delivery.’ GDNF has been available for treatment of PD for 30 years, but has never been approved for PD owing to inadequate brain delivery. Etanercept is used widely to treat inflammation in peripheral organs, but has never been developed for PD owing to lack of BBB transport of this decoy receptor. Therapeutic antibodies directed against α-synuclein have failed in clinical trials of PD (Lang et al., 2022; Pagano et al., 2022). This clinical trial failure is expected because therapeutic antibodies do not cross the BBB (Pardridge, 2023a). However, the issue of BBB transport of the α-synuclein antibodies is not even discussed in the reports of the failed clinical trials (Lang et al., 2022; Pagano et al., 2022), nor in the Editorial lamenting the failed clinical trials of the α-synuclein antibodies (Whone, 2022). A review of new therapeutic targets in PD makes no mention of how the new drugs will be delivered across the BBB (Soni et al., 2023). The development of small molecule drugs for PD is not a viable solution to the BBB problem for biologics, because 98% of all small molecules do not cross the BBB (Pardridge, 2022b). Going forward in PD drug development, biologics need to be re-engineered for BBB delivery, e.g., via the RMT pathway, which is the focus of this review. In the absence of viable solutions to the BBB drug delivery problem in PD drug development, one can anticipate the future centennial celebration of L-DOPA as the primary treatment of Parkinson’s disease.

## Author contributions

WP: Writing – original draft, Writing – review & editing.
